# Deciphering bidirectional causal links between oxidative stress and lung cancer risk through Mendelian randomization

**DOI:** 10.1007/s12672-025-03289-2

**Published:** 2025-07-28

**Authors:** XueLian Chen, JianRong Zeng, FengYun Cao, Jun Cai, Xin Ma, ChangYu Pu

**Affiliations:** 1 Department of Oncology, Suining City Traditional Chinese Medicine Hospital, Suining, Sichuan China; 2 Department of Oncology, Shehong City Traditional Chinese Medicine Hospital, Shehong, Sichuan China; 3 Department of Oncology, Bazhong City Traditional Chinese Medicine Hospital, Bazhong, Sichuan China

**Keywords:** Mendelian randomization, Oxidative stress, Lung cancer, Albumin, Lactate, Monounsaturated fatty acids, Genome-wide association studies

## Abstract

**Background:**

Lung cancer remains the leading cause of cancer-related mortality globally. Despite advancements in treatment, survival rates for advanced-stage disease remain suboptimal, emphasizing the need for novel prevention strategies. Oxidative stress (OS), resulting from an imbalance between reactive oxygen species (ROS) and antioxidants, is implicated in lung cancer pathogenesis. This study aimed to explore bidirectional causal relationships between genetically predicted oxidative stress injury biomarkers (OSIBs) and lung cancer risk using mendelian randomization (MR).

**Methods:**

A two-sample bidirectional MR approach was used to assess causal effects of 16 OSIBs on lung cancer subtypes (small cell lung cancer, squamous cell carcinoma, and adenocarcinoma) and vice versa. Genetic data were derived from large-scale genome-wide association studies in European populations.

**Results:**

MR analysis revealed significant associations. Higher albumin levels were associated with reduced adenocarcinoma risk (OR = 0.599, 95%CI: 0.369–0.974, *P* = 0.039). Elevated monounsaturated fatty acids levels were linked to an increased risk of squamous cell carcinoma (OR = 1.742, 95% CI: 1.095–2.772, *P* = 0.019). Increased lactate levels were positively associated with small cell lung cancer (OR = 4.565, 95% CI: 1.009–20.657, *P* = 0.049). Reverse MR analysis did not suggest causal effects of lung cancer on OSIBs.

**Conclusion:**

These findings highlight the distinct roles of OSIBs in lung cancer risk and underscore oxidative stress's pivotal role in cancer development. Further research is needed to validate these biomarkers for early detection and preventive strategies.

**Supplementary Information:**

The online version contains supplementary material available at 10.1007/s12672-025-03289-2.

## Introduction

Lung cancer remains the leading cause of cancer-related mortality worldwide, accounting for an estimated 2.2 million new cases (11.4%) and 1.8 million deaths (18.0%) in 2020 [1]. It is a highly heterogeneous disease, encompassing a range of histological subtypes, with the most common being non-small cell lung cancer and small cell lung cancer, non-small cell lung cancer is further divided into three major subtypes: adenocarcinoma, squamous cell carcinoma, and large cell carcinoma, with adenocarcinoma and squamous cell carcinoma accounting for the majority of cases [2, 3]. These subtypes differ not only in their histological characteristics but also in their molecular profiles and clinical outcomes, which complicates treatment strategies [4]. Despite significant advances in lung cancer treatment, including the development of targeted therapies (e.g., EGFR inhibitors) and immunotherapies (e.g., PD-1 inhibitors), survival rates, particularly for advanced stages of the disease, remain alarmingly low [5, 6]. The 5-year survival rate for stage IV lung cancer is approximately 19%,, underscoring the urgent need for better preventive and therapeutic approaches [7]. Identifying modifiable risk factors, especially those that can be targeted for early intervention or prevention, is crucial for improving patient outcomes.

Oxidative stress (OS), which arises from an imbalance between the production of reactive oxygen species (ROS) and the body’s antioxidant defenses, has long been implicated in the pathogenesis of lung cancer [8]. ROS can cause DNA damage, protein oxidation, and lipid peroxidation, all of which contribute to carcinogenesis [9, 10]. Several oxidative stress injury biomarkers (OSIBs) have been identified and associated with various types of cancer, including lung cancer. For example, increased levels of lipid peroxidation products such as malondialdehyde and 4-hydroxynonenal have been linked to higher lung cancer risk, particularly in smokers [11, 12]. Similarly, decreased levels of antioxidants such as glutathione and vitamin C, which play key roles in neutralizing ROS, have been observed in lung cancer patients [13]. Additionally, plasma albumin a major antioxidant protein, has been inversely associated with lung cancer risk, suggesting its potential as a protective factor [14, 15]. Furthermore, zinc, which is involved in maintaining antioxidant enzyme activity, has been identified as a potential biomarker for lung cancer risk. A study by Bai et al. demonstrated that low zinc levels in plasma were correlated with an increased risk of lung cancer [16]. However, despite these associations, observational studies on OSIBs and lung cancer are often confounded by factors such as smoking, environmental pollutants, and other comorbidities, which complicate the ability to establish direct causal relationships between these biomarkers and lung cancer [17].

To address this limitation, Mendelian randomization (MR) has emerged as a powerful tool to assess causal effects by using genetic variants as instrumental variables [18]. MR offers an advantage over traditional observational studies by helping to reduce confounding and reverse causation [19]. In this study, we employed a two-sample bidirectional MR approach to investigate the causal relationships between genetically predicted OSIBs and lung cancer risk. Using genetic summary data from large-scale genome-wide association studies (GWAS), we assessed the effects of OSIBs on different subtypes of lung cancer, as well as the potential reverse causal relationships. By utilizing a conservative selection of instrumental variables and performing rigorous sensitivity analyses, we aimed to provide more reliable and unbiased estimates of these causal associations.

The significance of this study lies in its ability to clarify the role of OS in lung cancer etiology, as well as to explore the possibility that lung cancer itself could influence OSIBs. The findings from this study not only contribute to the understanding of lung cancer pathophysiology but also hold potential for identifying novel biomarkers for lung cancer risk prediction. Additionally, this research may inform future therapeutic strategies, particularly those aimed at modulating OS as a means of cancer prevention or treatment.

## Methods

### Study design

**Fig. 1:**
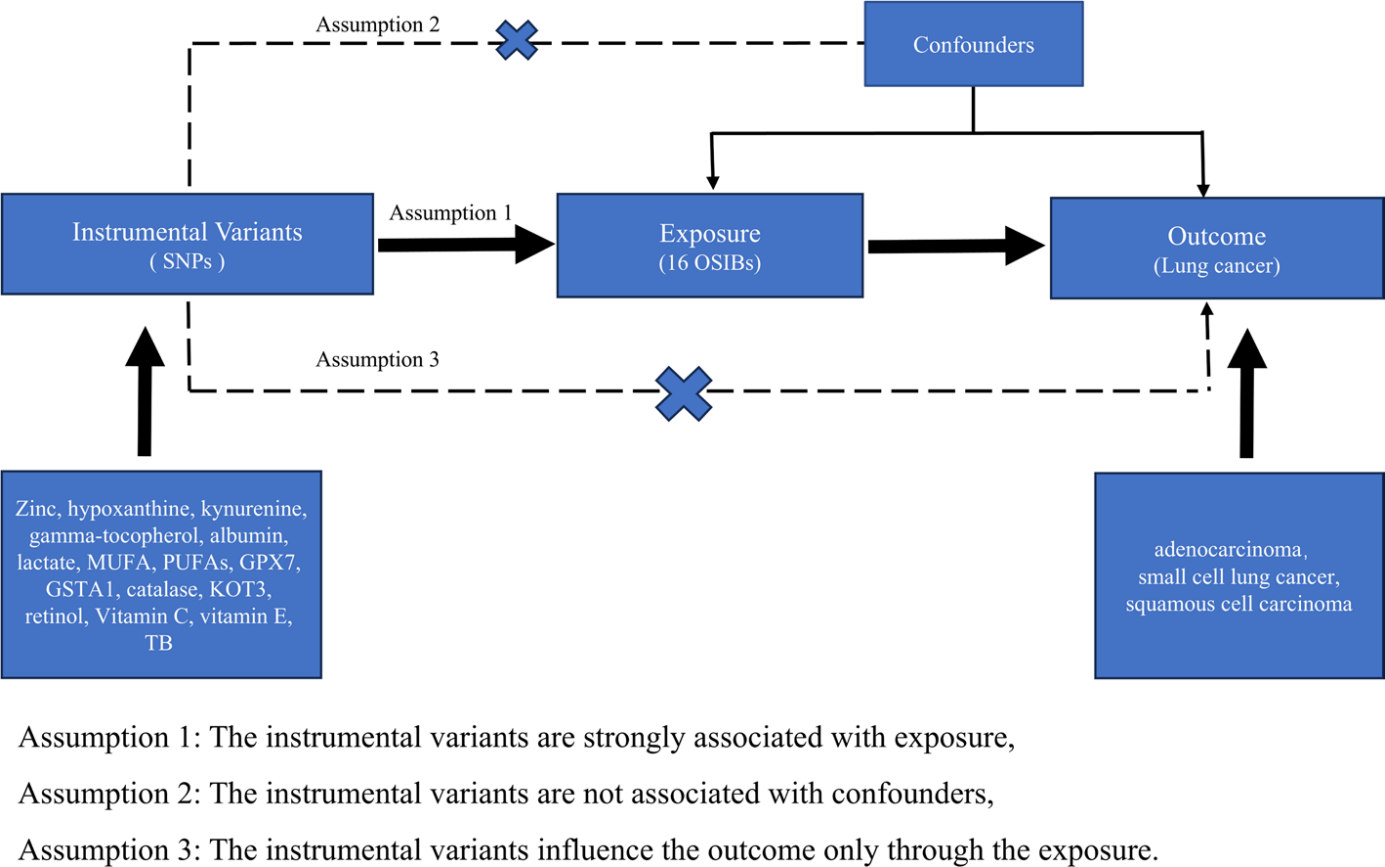
Overview of the Mendelian randomization (MR) design

This study employed a two-sample MR bidirectional study using single‐nucleotide polymorphisms (SNPs) to assess the causal association of sixteen OSIBs with three lung cancer. A group of OS injury biomarkers was composed of Zinc, hypoxanthine, kynurenine, gamma-tocopherol, albumin, lactate, monounsaturated fatty acids (MUFAs), polyunsaturated fatty acids (PUFAs), glutathione peroxidase 7 (GPX), glutathione S-transferase A1 (GST), catalase, kynurenine–oxoglutarate transaminase 3 (KOT3), retinol, Vitamin C, vitamin E, total bilirubin (TB); lung cancer included small cell lung cancer, non-small cell lung cancer, squamous, non-small cell lung cancer, adenocarcinoma(Table 1). MR relies on three assumptions: (1) the genetic variants are strongly associated with the exposure (relevance); (2) they are independent of confounders (independence); and (3) they influence the outcome only through the exposure (exclusivity) (Fig. 1). Our study included independent GWAS cohorts of European ancestry with no overlap. Ethical approval was not required because the study was based on existing publications and public databases.

**Table 1:** Detailed information regarding studies and datasets used in the present study

Exposure or outcome	GWAS ID	Participants	Ancestry	PMID
Zinc	ieu-a-1079	2,603	European	23,720,494
Hypoxanthine	met-a-361	7,287	European	24,816,252
Kynurenine	met-a-375	7,816	European	24,816,252
Gamma-tocopherol	met-a-571	6,226	European	24,816,252
Albumin	met-d-Albumin	115,060	European	NA
Lactate	met-d-Lactate	114,802	European	NA
MUFA	met-d-MUFA	114,999	European	NA
PUFA	met-d-PUFA	114,999	European	NA
GPX7	prot-a-1265	3,301	European	29,875,488
GSTA1	prot-a-1283	3,301	European	29,875,488
Catalase	prot-a-367	3,301	European	29,875,488
KOT3	prot-a-378	3,301	European	29,875,488
Retinol	ukb-b-17,406	62,991	European	NA
Vitamin C	ukb-b-19,390	64,979	European	NA
Vitamin E	ukb-b-6888	64,979	European	NA
TB	ukb-d-30840_raw	NA	European	NA
Small cell lung cancer	finn-b-C3_SCLC	218,792	European	NA
Squamous cell carcinoma	finn-b-C3_NSCLC_SQUAM	218,792	European	NA
Adenocarcinoma	finn-b-C3_NSCLC_ADENO	218,792	European	NA

MUFA, monounsaturated fatty acid; PUFA, polyunsaturated fatty acid; GPX, glutathione peroxidase; GST, glutathione S-transferase; KOT3, kynurenine–oxoglutarate transaminase 3; TB, total bilirubin

### Genetic associations with oxidative stress injury biomarkers

We gathered summary data from a range of published GWAS studies involving European ancestry populations, focusing on 16 OSIBs across various metabolic pathways. Specifically, the data for Zinc were obtained from Evans’ study [20], while hypoxanthine, kynurenine, and gamma-tocopherol were drawn from the work of Shin [21]. Similarly, Sun’s research provided insights into GPX, GST, catalase, and KOT3 [22]. Biomarkers such as albumin, lactate, MUFA, and PUFA were sourced from Borges’ study, further enriching our dataset. The remaining biomarkers were extracted from the extensive dataset of the UK Biobank. This diverse and meticulously curated collection of data underscores the comprehensive nature of our investigation.

### Genetic associations with lung cancer

The genetic association data for lung cancer subtypes in this study were obtained from the FinnGen project, a comprehensive genetic research initiative in Finland. The FinnGen database links genetic data from over 500,000 participants with detailed health information from national registries, enabling in-depth genetic analyses across a wide range of diseases. Specifically, data on small cell lung cancer, squamous cell carcinoma, and adenocarcinoma were included in our analysis. FinnGen’s large-scale, population-based approach provides valuable insights into the genetic underpinnings of these lung cancer subtypes, offering a unique resource for exploring complex genetic interactions and disease mechanisms.

### The selection of IVs

SNPs significantly associated with OSIBs were identified using a stringent P-value threshold of < 1e-6, ensuring that only robust genetic instruments were selected for the Mendelian randomization (MR) analyses. To minimize the potential for pleiotropy, SNPs in high linkage disequilibrium (LD) were excluded, with an LD threshold of r² < 0.001 within a 10,000-kb window. This step ensures that genetic variants used as instrumental variables (IVs) are independent, reducing the risk of confounding due to correlated genetic effects. In addition, only SNPs with F-statistics greater than 10 were retained to avoid weak instrument bias, a critical step to ensure the validity of causal inferences. To further reduce ambiguity, palindromic SNPs—those with identical allele pairs (e.g., A/T or G/C) in both the exposure and outcome traits—were excluded to prevent issues with allele orientation, ensuring consistent interpretation of genetic associations across both exposure and outcome.

The same rigorous criteria were applied to the reverse MR analysis, where OSIBs and lung cancer were alternatively treated as the exposure or outcome. This bidirectional approach involved conducting 96 distinct MR studies, with each biomarker and lung cancer subtype analyzed in both directions, allowing for a comprehensive evaluation of potential causal relationships between OSIBs and lung cancer. By using this robust and conservative strategy, we aimed to minimize confounding, maximize the strength of the instrumental variables, and provide reliable causal estimates in both directions.

### Statistical analyses

In the primary MR analyses investigating the causal relationship between OSIBs and lung cancer, five MR methods were employed: MR Egger, weighted median, inverse variance weighted (IVW), simple mode, and weighted mode. The IVW method, which is particularly powerful when all genetic variants serve as valid instrumental variables (IVs), was used as the primary approach to estimate causal effects, as it provides the most precise estimates under the assumption of no horizontal pleiotropy [23]. MR Egger regression, with its ability to detect and correct for horizontal pleiotropy, was used as a complementary method, while the weighted median and mode-based methods, which offer robustness against pleiotropy, were also included to validate the findings. To account for the potential inflation of type I error due to multiple testing, false discovery rate (FDR) correction was applied to the MR results as a sensitivity analysis rather than the primary criterion for causal inference—a practice commonly adopted in exploratory MR studies, where the primary goal is to identify potential causal relationships for further investigation rather than to draw definitive conclusions. Heterogeneity among the instrumental variables was assessed using Cochrane’s Q-test, with a p-value greater than 0.05 indicating no significant heterogeneity [24]. Additionally, horizontal pleiotropy was examined using the MR-Egger intercept test, where a non-zero intercept suggests the presence of pleiotropic effects, potentially confounding the causal inference [25].

All statistical analyses and data visualizations were performed using R (version 4.3.2), with the “TwoSampleMR” R package used for conducting univariable MR analyses. The study’s findings were reported in accordance with the STROBE-MR (Strengthening the Reporting of Mendelian Randomization Studies) guidelines, which aim to improve the transparency and reproducibility of MR studies by promoting standardized reporting of methods, results, and assumptions [26].

## Results

### Determination of instrumental variables

Summary data of SNP-phenotype associations were obtained from GWAS for each phenotype. All instruments had an F-statistics of > 19, which is above the standard cut-of (> 10) indicating sufficient instrumental strength (Tables S1-S2).

### The causal effect of genetically predicted OSIBs on lung cancer

**Fig. 2:**
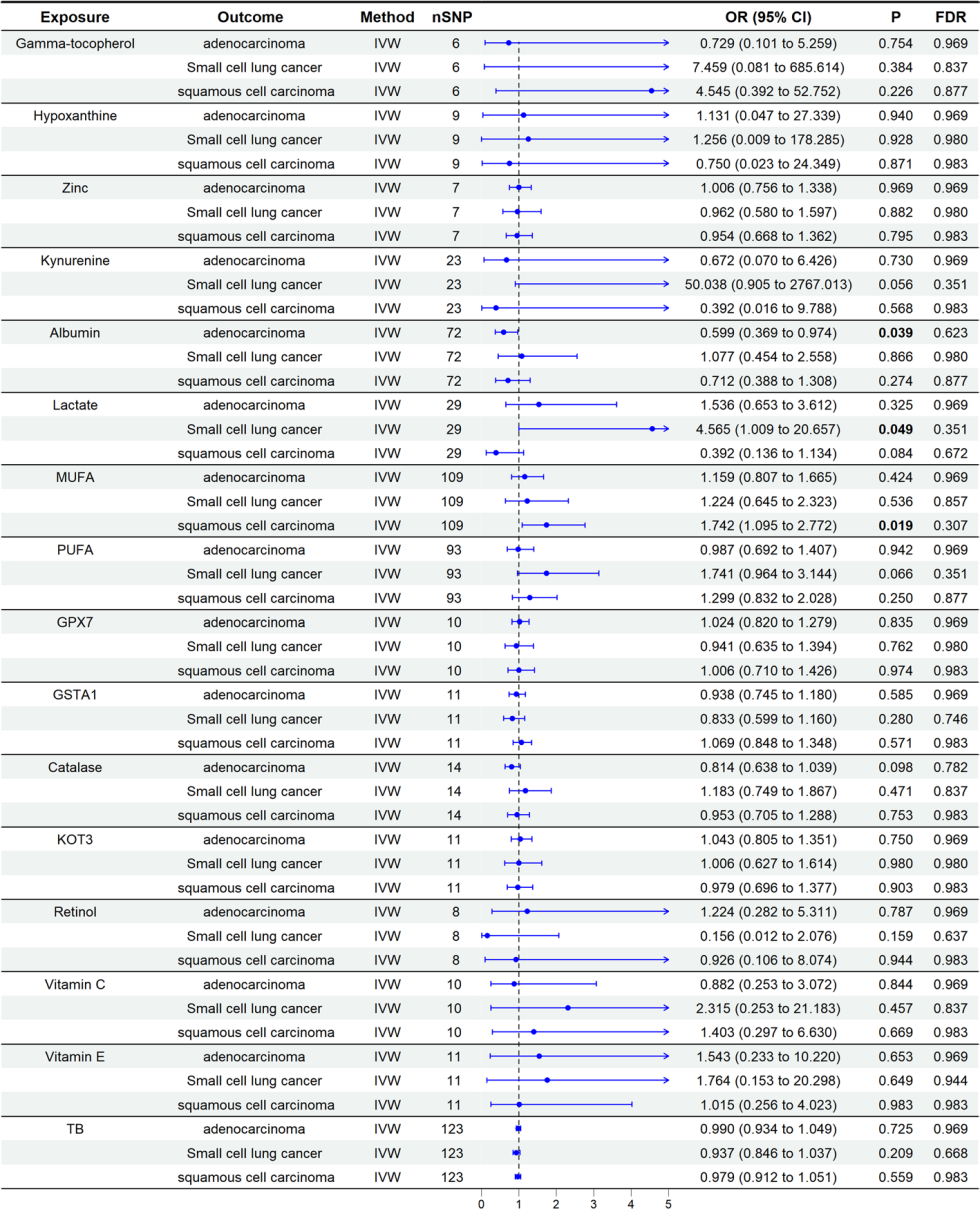
A forest plot showing associations between genetically determined 16 OSIBs and lung cancer subtypes based on IVW MR analysis. OSIB, oxidative stress injury biomarker; MUFA, monounsaturated fatty acid; PUFA, polyunsaturated fatty acid; GPX, glutathione peroxidase; GSTA1, glutathione S-transferase A1; KOT3, kynurenine–oxoglutarate transaminase 3; TB, total bilirubin; MR, mendelian randomization; SNP, single nucleotide polymorphism; IVW, inverse-variance weighted, OR, odds ratio; CI, confidence interval

**Fig. 3:**
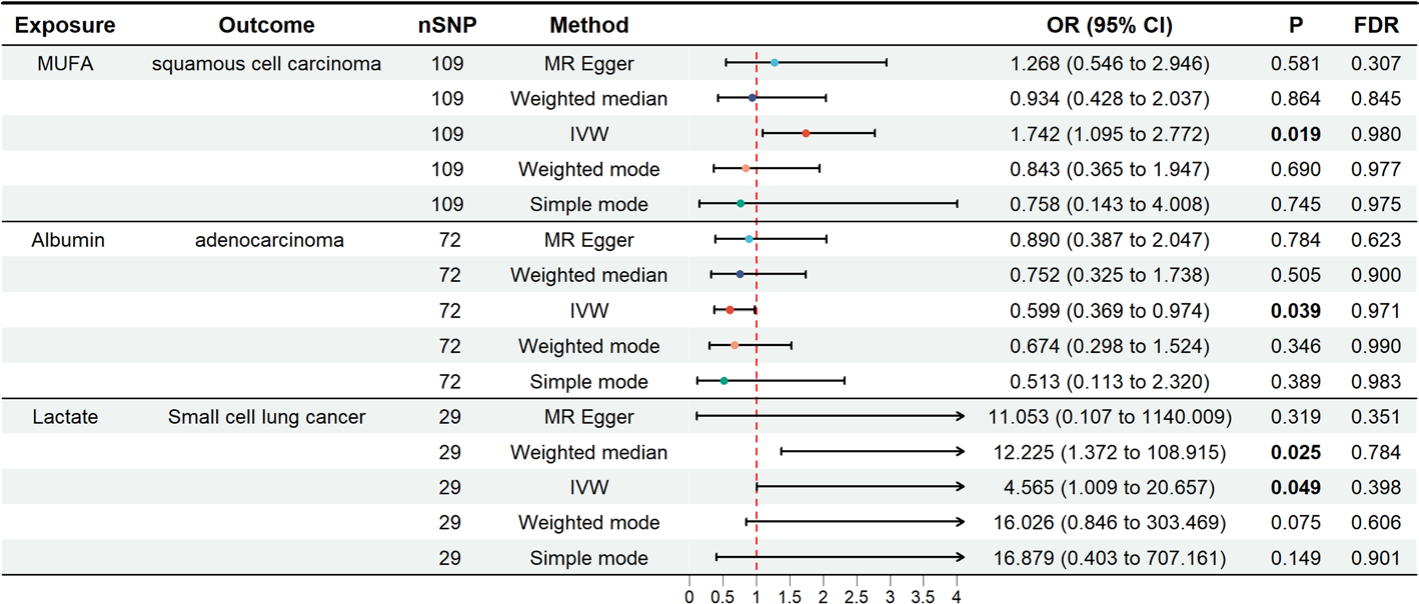
A forest plot of genetically predicted OSIBs and their associations with lung cancer subtypes: focus on MUFA, albumin, and lactate. OSIB, oxidative stress injury biomarker; MUFA, monounsaturated fatty acid; MR, mendelian randomization; SNP, single nucleotide polymorphism, IVW inverse-variance weighted, OR odds ratio, CI confidence interval

The two-sample MR analysis assessed the causal effects of genetically predicted OSIBs on lung cancer risk. The results indicated significant associations between genetically predicted levels of several OSIBs and different subtypes of lung cancer (Fig. 2). Specifically, higher levels of Albumin were associated with a reduced risk of adenocarcinoma (OR = 0.599, 95% CI: 0.369–0.974, *P* = 0.039). In contrast, higher levels of MUFA were linked to an increased risk of squamous cell carcinoma (OR = 1.742, 95% CI: 1.095–2.772, *P* = 0.019). Additionally, elevated Lactate levels were associated with a higher risk of small cell lung cancer (OR = 4.565, 95% CI: 1.009–20.657, *P* = 0.049) (Fig. 3). Further details on the results of the additional forward MR can be found in Supplementary Figure S1-S3.

Sensitivity analyses, including MR-Egger intercept tests, indicated no significant evidence of pleiotropy, with p-values of 0.257 for Albumin on adenocarcinoma, 0.378 for MUFA on squamous cell carcinoma, and 0.696 for Lactate on small cell lung cancer. Furthermore, tests for heterogeneity, including Cochran’s Q statistic and Rucker’s Q test, did not show significant results (*P* > 0.05), suggesting that the associations were not influenced by confounding factors (Table 2).

**Table 2:** Heterogeneity and pleiotropy analysis in MR analysis

Exposure	Outcome	Heterogeneity (MR Egger) p_value	Heterogeneity (IVW) p_value	Egger intercept	Pleiotropy p_value
Gamma-tocopherol	Squamous cell carcinoma	0.873	0.516	0.265	0.125
Hypoxanthine		0.926	0.961	−0.009	0.958
Zinc		0.260	0.150	−0.089	0.076
Kynurenine		0.543	0.639	−0.088	0.612
Albumin		0.539	0.539	−0.019	0.326
Lactate		0.569	0.611	0.026	0.649
MUFA		0.302	0.306	0.015	0.378
PUFA		0.155	0.157	0.018	0.358
GPX7		0.255	0.110	0.135	0.106
GSTA1		0.966	0.932	0.086	0.274
Catalase		0.927	0.861	0.112	0.188
KOT3		0.559	0.216	−0.124	0.045
Retinol		0.172	0.192	0.095	0.466
Vitamin C		0.567	0.665	−0.008	0.913
Vitamin E		0.613	0.611	0.062	0.353
TB		0.812	0.818	−0.007	0.433
Gamma-tocopherol	Adenocarcinoma	0.508	0.603	−0.058	0.636
Hypoxanthine		0.190	0.242	0.081	0.628
Zinc		0.474	0.522	−0.017	0.633
Kynurenine		0.749	0.779	0.096	0.498
Albumin		0.820	0.811	−0.018	0.257
Lactate		0.565	0.571	−0.042	0.360
MUFA		0.641	0.462	0.036	0.007
PUFA		0.201	0.183	0.021	0.181
GPX7		0.935	0.875	−0.065	0.253
GSTA1		0.155	0.131	−0.081	0.288
Catalase		0.955	0.931	−0.075	0.276
KOT3		0.246	0.314	0.015	0.758
Retinol		0.619	0.605	−0.081	0.351
Vitamin C		0.720	0.629	−0.071	0.224
Vitamin E		0.001	0.001	0.044	0.638
TB		0.389	0.413	−0.001	0.887
Gamma-tocopherol	Small cell lung cancer	0.570	0.691	−0.039	0.857
Hypoxanthine		0.824	0.877	0.097	0.695
Zinc		0.401	0.451	0.026	0.685
Kynurenine		0.240	0.138	−0.384	0.223
Albumin		0.892	0.903	−0.015	0.599
Lactate		0.877	0.899	−0.031	0.696
MUFA		0.880	0.893	−0.001	0.969
PUFA		0.547	0.574	−0.007	0.800
GPX7		0.808	0.811	−0.081	0.412
GSTA1		0.908	0.938	0.042	0.702
Catalase		0.502	0.333	0.209	0.095
KOT3		0.268	0.261	0.085	0.339
Retinol		0.635	0.645	0.127	0.402
Vitamin C		0.666	0.748	−0.028	0.776
Vitamin E		0.080	0.116	−0.021	0.866
Total bilirubin		0.790	0.808	−0.002	0.881

MR, mendelian randomization; IVW, Inverse variance weighted; MUFA, monounsaturated fatty acid; PUFA, polyunsaturated fatty acid; GPX, glutathione peroxidase; GST, glutathione S-transferase; KOT3, kynurenine--oxoglutarate transaminase 3; TB, total bilirubin

### The causal effect of genetically predicted lung cancer on OSIBs

To investigate the potential causal effect of genetically predicted lung cancer on 16 OSIBs, reverse MR analysis was performed. The analysis found no significant causal associations between genetically predicted squamous cell carcinoma, adenocarcinoma, or small cell lung cancer and any of the 16 OSIBs studied. For instance, Albumin showed no effect on adenocarcinoma (OR = 1.003, 95% CI: 0.996–1.011, *P* = 0.380), Cat showed no effect on squamous cell carcinoma (OR = 1.012, 95% CI: 0.984–1.040, *P* = 0.411), and Lactate had no impact on small cell lung cancer (OR = 0.998, 95% CI: 0.991–1.005, *P* = 0.590) (Tables S3-S5).

Sensitivity analyses, including MR-Egger regression and other methods, revealed no evidence of significant pleiotropy (*P* > 0.05), confirming the robustness of the findings. Furthermore, most of the study results showed no heterogeneity, supporting the consistency of the causal estimates (Tables S6). Taken together, these findings indicate that genetically predicted lung cancer does not have a causal effect on the 16 OSIBs considered in this study.

## Discussion

In this study, we conducted a two-sample MR analysis to explore the causal relationship between OSIBs and lung cancer risk. Our findings suggest that certain OSIBs, such as Albumin, MUFA, and Lactate, are significantly associated with lung cancer risk. Higher genetically predicted Albumin levels were associated with a reduced risk of adenocarcinoma, while elevated MUFA levels were linked to an increased risk of squamous cell carcinoma. Additionally, higher Lactate levels were positively correlated with small cell lung cancer risk. These results highlight the distinct roles of OSIBs in the development of different lung cancer subtypes.

It is worth noting, however, that none of these associations remained statistically significant after FDR correction. Nonetheless, this does not necessarily negate their potential biological relevance. In exploratory MR studies, FDR correction is typically used as a supplementary approach to support the robustness of the findings, rather than as a definitive filter—especially when exposures are correlated and the primary aim is to generate hypotheses for further validation.

These findings are consistent with existing literature on the role of OS in cancer development. OS, caused by the accumulation of ROS, is a well-established factor in the initiation and progression of various cancers, including lung cancer [13]. ROS induce DNA mutations, protein misfolding, and lipid peroxidation, which can all contribute to carcinogenesis [27]. Albumin, a major plasma protein with antioxidant properties, is believed to mitigate oxidative damage [28]. The protective effect of Albumin against adenocarcinoma risk observed in our study may be due to its role in buffering OS and maintaining cellular homeostasis. Lower levels of albumin in cancer patients have been linked to poorer prognosis, possibly because reduced antioxidant capacity allows for increased oxidative damage and tumor progression [29]. Adenocarcinoma, which is often associated with environmental and genetic factors such as smoking and mutations in EGFR and ALK genes, may be more susceptible to the protective effects of Albumin, which helps maintain cellular integrity in the presence of OS [30]. Our findings suggest that albumin may reduce adenocarcinoma risk by preventing DNA damage and inflammatory responses that are critical for tumor initiation and metastasis.

MUFAs, although often considered beneficial for cardiovascular health [31], can contribute to cancer development under certain conditions, particularly in lung cancer. MUFAs influence OS, inflammation, and lipid metabolism—factors central to cancer pathogenesis [32, 33]. High MUFA intake has been associated with increased lipid peroxidation, which leads to the production of reactive aldehydes and other toxic compounds that promote cancer cell proliferation and metastasis [34]. Additionally, MUFAs have been shown to alter tumor microenvironmental factors, such as inflammation and immune cell activation, that favor tumor progression. The increased risk of squamous cell carcinoma associated with higher genetically predicted MUFA levels in our study is consistent with these mechanisms. Squamous cell carcinoma often linked to smoking and environmental exposures, may be particularly responsive to altered lipid metabolism, making it more sensitive to the pro-inflammatory and oxidative effects of MUFAs [35, 36]. Our findings suggest that MUFAs could exacerbate squamous cell carcinoma development by enhancing oxidative damage and inflammatory pathways that promote tumorigenesis in this specific subtype.

The positive association between Lactate and small cell lung cancer risk is particularly noteworthy. Lactate, a byproduct of anaerobic metabolism in tumors, plays a crucial role in the Warburg effect, a hallmark of cancer metabolism [37]. In small cell lung cancer, which is characterized by its rapid growth and aggressive behavior, cells primarily rely on glycolysis even in the presence of oxygen, producing large amounts of Lactate [37, 38] Lactate not only serves as an energy substrate for proliferating cancer cells but also promotes tumor progression through several mechanisms [39]. It can stimulate angiogenesis, immune suppression, and metastasis—all of which are vital processes for aggressive cancer phenotypes like small cell lung cancer [37, 40]. Our study identified a genetically predicted positive association between lactate levels and small cell lung cancer risk. However, this result should be interpreted with caution, as the odds ratio was relatively high (OR = 4.565) and the confidence interval was wide (95% CI: 1.009–20.657), indicating substantial uncertainty and potential statistical instability. The wide confidence interval may reflect limited power or variability in the instrumental variables for lactate. Therefore, while this association is biologically plausible and aligns with existing knowledge of tumor metabolism, it requires validation in future independent studies with greater statistical power.Taken together, our findings suggest that lactate may contribute to the pathogenesis of small cell lung cancer through its metabolic functions. Nonetheless, these results are exploratory in nature and should not be overinterpreted. Further mechanistic and epidemiological studies are needed to confirm these observations and clarify the role of lactate in small cell lung cancer.

Conversely, other OSIBs, such as Hxa and Toc, did not show significant associations with lung cancer risk in our study. This could be explained by their differing roles in OS and metabolism across cancer subtypes. For example, Hxa, a purine metabolite, may not be directly involved in the OS pathways that drive lung cancer development, or its effects could be overshadowed by other more prominent biomarkers. Similarly, Toc, a form of vitamin E, acts as an antioxidant but may not have a strong impact on the pathophysiology of lung cancer compared to other biomarkers like albumin, MUFAs, or lactate. The lack of significant association between these OSIBs and lung cancer could reflect the complex nature of cancer metabolism, where a variety of factors interact to influence disease progression. Additionally, genetic variability in OS regulation may lead to differential associations across cancer subtypes, indicating the need for further research to explore how these biomarkers interact with genetic and environmental factors that influence lung cancer risk.

This study has several strengths, including the use of Mendelian randomization, which reduces biases typically found in observational studies, such as confounding and reverse causality. The bidirectional design of our MR analysis also allows us to assess potential reverse causation, an important consideration in complex diseases like cancer. The use of large-scale summary genetic data increases the statistical power and generalizability of our findings. However, there are several limitations. While MR is a powerful tool for causal inference, it assumes that genetic variants are not associated with confounders other than the exposure of interest. Despite rigorous quality control, horizontal pleiotropy—where genetic variants influence the outcome via alternative pathways—remains a potential concern. In addition, the genetic variants used were mainly derived from European populations, which may limit the generalizability of our findings to other ethnic groups. Future studies in more diverse populations are warranted. Our analysis was also restricted to the availability and scope of existing GWAS summary data, which limits the completeness of information on clinical characteristics, such as cancer staging, treatment strategies, and survival outcomes. Consequently, stratified or subgroup analyses could not be performed. Moreover, because the exposure and outcome data were obtained from different sources and potentially collected during different time periods, temporal alignment could not be fully ensured, which may affect the interpretation of causal estimates. Furthermore, although a range of associations were explored, none remained statistically significant after correction for multiple testing. While this raises concerns about potential false positives, it also highlights the conservative nature of such corrections, particularly in the context of correlated exposures. Lastly, the use of summary-level data precluded any investigation of interactions, mediation effects, or the role of time-varying exposures, which are important in clinical and epidemiological contexts.

In conclusion, our study provides evidence for a causal relationship between OSIBs and lung cancer risk. Specifically, higher albumin levels were associated with a reduced risk of adenocarcinoma, while elevated MUFA and lactate levels were linked to an increased risk of squamous cell carcinoma and small cell lung cancers, respectively. These findings underscore the importance of OS in lung cancer pathogenesis and suggest potential biomarkers for early detection and targeted prevention strategies. Further research is needed to explore the clinical relevance of these biomarkers and to investigate the underlying biological mechanisms in more detail.

## Supplementary Information

Supplementary Material 1

## Data Availability

All the Mendelian randomization study files are available from GWAS. (URL https://gwas.mrcieu.ac.uk)

## Abbreviations

OS: Oxidative stress
ROS: Reactive oxygen species
OSIB: Oxidative stress injury biomarker
MR: Mendelian randomization
GWAS: Genome-wide association studies
SNP: Single-nucleotide polymorphism
MUFA: Monounsaturated fatty acid
PUFA: Polyunsaturated fatty acid
GPX: Glutathione peroxidase
GST: Glutathione S-transferase
KOT3: Kynurenine–oxoglutarate transaminase 3
TB: Total bilirubin
LD: Linkage disequilibrium
IV: Instrumental variable
IVW: Inverse variance weighted
OR: Odds ratio
CI: Confidence interval
SE: Standard error
