# Supplementary material for: Deciphering bidirectional causal links between oxidative stress and lung cancer risk through Mendelian randomization
Source: Discov Oncol. 2025 Jul 28;16:1421. doi: 10.1007/s12672-025-03289-2 (PMC12304326; doi:10.1007/s12672-025-03289-2)
Supplement: Supplementary file 1 — Supplementary Material 1 [file 12672_2025_3289_MOESM1_ESM.zip › Supplementary materials/Supplementary materials.docx]

***Supplementary Materials***

**SUPPLEMENTARY NOTES**

**Table S1.** Characteristics of SNPs associated with 16 OSIBs.

**Table S2.** Characteristics of SNPs associated with lung cancer.

**Table S3.** Associations between genetically predicted adenocarcinoma and 16 OSIBs.

**Table S4.** Associations between genetically predicted squamous and 16 OSIBs.

**Table S5.** Associations between genetically predicted small cell lung cancer and 16 OSIBs.

**Table S6.** Heterogeneity and horizontal pleiotropy of lung cancer and 16 OSIBs.

| **Table S1. Characteristics of SNPs associated with 16 OSIBs.** | | | | | | | | | | |
| --- | --- | --- | --- | --- | --- | --- | --- | --- | --- | --- |
| **Albumin** | | | | | | | | | | |
| **SNP** | **EA** | **Position** | **EAF** | **BETA** | **SE** | **P** | **N** | **R2** | **F** | **palindromic** |
| rs28929474 | T | 94844947 | 0.020 | 0.429 | 0.015 | 7.29E-185 | 115060 | 7.25E-03 | 840.77 | FALSE |
| rs17580 | A | 94847262 | 0.048 | 0.145 | 0.010 | 7.20E-51 | 115060 | 1.95E-03 | 225.04 | TRUE |
| rs10419198 | T | 50038017 | 0.253 | -0.056 | 0.005 | 2.70E-32 | 115060 | 1.21E-03 | 139.96 | FALSE |
| rs58895965 | A | 35551428 | 0.175 | 0.063 | 0.005 | 3.50E-31 | 115060 | 1.17E-03 | 134.86 | FALSE |
| rs77542162 | G | 67081278 | 0.023 | -0.156 | 0.014 | 2.80E-29 | 115060 | 1.10E-03 | 126.15 | FALSE |
| rs1260326 | C | 27730940 | 0.604 | -0.044 | 0.004 | 7.40E-26 | 115060 | 9.60E-04 | 110.56 | FALSE |
| rs6871112 | G | 72125370 | 0.540 | -0.039 | 0.004 | 2.10E-21 | 115060 | 7.84E-04 | 90.29 | TRUE |
| rs139974673 | C | 44027885 | 0.026 | 0.121 | 0.013 | 1.90E-20 | 115060 | 7.46E-04 | 85.89 | FALSE |
| rs1461729 | G | 9187242 | 0.899 | 0.060 | 0.007 | 1.70E-18 | 115060 | 6.69E-04 | 77.05 | FALSE |
| rs9912287 | A | 1630992 | 0.219 | 0.042 | 0.005 | 1.20E-16 | 115060 | 5.96E-04 | 68.58 | FALSE |
| rs3740688 | T | 47380340 | 0.545 | 0.032 | 0.004 | 8.50E-15 | 115060 | 5.23E-04 | 60.22 | FALSE |
| rs114949263 | C | 150498245 | 0.111 | 0.050 | 0.007 | 3.30E-14 | 115060 | 5.00E-04 | 57.52 | FALSE |
| rs74892229 | A | 16854480 | 0.102 | -0.051 | 0.007 | 4.60E-14 | 115060 | 4.94E-04 | 56.91 | FALSE |
| rs34754216 | T | 161575538 | 0.424 | -0.030 | 0.004 | 1.80E-12 | 115060 | 4.32E-04 | 49.68 | FALSE |
| rs11128594 | G | 12303417 | 0.194 | -0.036 | 0.005 | 6.20E-12 | 115060 | 4.11E-04 | 47.26 | FALSE |
| rs61653336 | A | 40297658 | 0.165 | -0.037 | 0.006 | 3.20E-11 | 115060 | 3.83E-04 | 44.04 | FALSE |
| rs13108218 | G | 3443931 | 0.615 | -0.028 | 0.004 | 4.60E-11 | 115060 | 3.77E-04 | 43.36 | FALSE |
| rs57274629 | G | 66110292 | 0.358 | 0.029 | 0.004 | 4.60E-11 | 115060 | 3.76E-04 | 43.32 | FALSE |
| rs11589479 | A | 155033308 | 0.167 | 0.035 | 0.006 | 2.70E-10 | 115060 | 3.46E-04 | 39.86 | FALSE |
| rs4886992 | C | 78325229 | 0.206 | -0.032 | 0.005 | 5.70E-10 | 115060 | 3.34E-04 | 38.41 | FALSE |
| rs55881006 | A | 74222782 | 0.034 | -0.070 | 0.011 | 1.20E-09 | 115060 | 3.21E-04 | 36.91 | FALSE |
| rs3768321 | T | 40035928 | 0.197 | -0.031 | 0.005 | 1.50E-09 | 115060 | 3.18E-04 | 36.56 | FALSE |
| rs8107347 | A | 18612748 | 0.350 | -0.026 | 0.004 | 2.80E-09 | 115060 | 3.07E-04 | 35.29 | FALSE |
| rs58546652 | T | 247601778 | 0.628 | -0.025 | 0.004 | 3.10E-09 | 115060 | 3.05E-04 | 35.14 | FALSE |
| rs6734238 | G | 113841030 | 0.403 | -0.025 | 0.004 | 4.90E-09 | 115060 | 2.97E-04 | 34.21 | FALSE |
| rs34284056 | A | 60203855 | 0.275 | 0.027 | 0.005 | 6.50E-09 | 115060 | 2.93E-04 | 33.68 | FALSE |
| rs2933243 | A | 56860577 | 0.183 | 0.030 | 0.005 | 1.40E-08 | 115060 | 2.80E-04 | 32.23 | FALSE |
| rs648997 | T | 111982813 | 0.258 | 0.027 | 0.005 | 3.20E-08 | 115060 | 2.66E-04 | 30.60 | FALSE |
| rs10069690 | T | 1279790 | 0.259 | 0.026 | 0.005 | 5.20E-08 | 115060 | 2.58E-04 | 29.65 | FALSE |
| rs1791936 | A | 72877964 | 0.604 | 0.023 | 0.004 | 6.40E-08 | 115060 | 2.54E-04 | 29.23 | FALSE |
| rs72842819 | C | 7328821 | 0.121 | 0.034 | 0.006 | 7.20E-08 | 115060 | 2.52E-04 | 29.02 | FALSE |
| rs112026770 | A | 16562106 | 0.148 | -0.031 | 0.006 | 8.50E-08 | 115060 | 2.49E-04 | 28.70 | FALSE |
| rs6935537 | C | 44054459 | 0.199 | -0.028 | 0.005 | 9.20E-08 | 115060 | 2.48E-04 | 28.54 | FALSE |
| rs2691584 | C | 17816817 | 0.414 | 0.022 | 0.004 | 1.10E-07 | 115060 | 2.45E-04 | 28.21 | FALSE |
| rs9855653 | C | 23588125 | 0.589 | -0.022 | 0.004 | 1.50E-07 | 115060 | 2.40E-04 | 27.57 | FALSE |
| rs10796927 | C | 154630498 | 0.285 | 0.024 | 0.005 | 1.50E-07 | 115060 | 2.39E-04 | 27.54 | FALSE |
| rs115744844 | C | 48632756 | 0.016 | 0.092 | 0.018 | 1.80E-07 | 115060 | 2.37E-04 | 27.25 | FALSE |
| rs4470390 | G | 62707481 | 0.768 | -0.026 | 0.005 | 1.90E-07 | 115060 | 2.36E-04 | 27.18 | FALSE |
| rs7599 | G | 36038390 | 0.631 | -0.022 | 0.004 | 2.00E-07 | 115060 | 2.35E-04 | 27.07 | FALSE |
| rs9389272 | A | 135459837 | 0.194 | 0.027 | 0.005 | 2.50E-07 | 115060 | 2.31E-04 | 26.58 | FALSE |
| rs6855246 | G | 103112470 | 0.080 | -0.041 | 0.008 | 2.60E-07 | 115060 | 2.31E-04 | 26.54 | FALSE |
| rs565840574 | G | 17848398 | 0.352 | 0.027 | 0.005 | 3.10E-07 | 115060 | 2.27E-04 | 26.16 | FALSE |
| rs73225023 | T | 121127914 | 0.085 | -0.038 | 0.007 | 3.90E-07 | 115060 | 2.24E-04 | 25.77 | FALSE |
| rs6519133 | C | 39096602 | 0.397 | 0.021 | 0.004 | 5.10E-07 | 115060 | 2.19E-04 | 25.24 | FALSE |
| rs79687284 | C | 214150821 | 0.035 | 0.057 | 0.011 | 5.60E-07 | 115060 | 2.18E-04 | 25.05 | TRUE |
| rs2116327 | A | 90228697 | 0.326 | 0.022 | 0.004 | 5.60E-07 | 115060 | 2.18E-04 | 25.04 | FALSE |
| rs10828249 | A | 21824727 | 0.343 | -0.022 | 0.004 | 6.50E-07 | 115060 | 2.15E-04 | 24.75 | FALSE |
| rs186663077 | T | 35718144 | 0.015 | 0.085 | 0.017 | 7.00E-07 | 115060 | 2.14E-04 | 24.62 | FALSE |
| rs555754 | A | 160769423 | 0.468 | -0.021 | 0.004 | 7.40E-07 | 115060 | 2.13E-04 | 24.50 | FALSE |
| rs1962003 | A | 22562691 | 0.702 | -0.022 | 0.005 | 7.50E-07 | 115060 | 2.13E-04 | 24.48 | FALSE |
| rs140567911 | C | 13204404 | 0.039 | -0.054 | 0.011 | 7.90E-07 | 115060 | 2.12E-04 | 24.39 | FALSE |
| rs2316307 | C | 117082987 | 0.622 | -0.021 | 0.004 | 8.00E-07 | 115060 | 2.12E-04 | 24.35 | FALSE |
| rs633683 | C | 118504742 | 0.598 | 0.021 | 0.004 | 8.60E-07 | 115060 | 2.10E-04 | 24.21 | FALSE |
| rs12433544 | A | 72754711 | 0.628 | 0.021 | 0.004 | 1.00E-06 | 115060 | 2.07E-04 | 23.84 | TRUE |
| rs137957801 | G | 29528417 | 0.084 | -0.037 | 0.008 | 1.10E-06 | 115060 | 2.07E-04 | 23.83 | FALSE |
| rs6939158 | C | 31299310 | 0.043 | -0.052 | 0.011 | 1.10E-06 | 115060 | 2.07E-04 | 23.79 | TRUE |
| rs2556569 | T | 44895326 | 0.049 | -0.048 | 0.010 | 1.10E-06 | 115060 | 2.07E-04 | 23.77 | FALSE |
| rs13079232 | G | 59564008 | 0.247 | -0.023 | 0.005 | 1.10E-06 | 115060 | 2.06E-04 | 23.76 | TRUE |
| rs59916403 | T | 242370751 | 0.352 | -0.021 | 0.004 | 1.10E-06 | 115060 | 2.06E-04 | 23.75 | FALSE |
| rs3749748 | T | 127350549 | 0.246 | -0.023 | 0.005 | 1.20E-06 | 115060 | 2.06E-04 | 23.66 | FALSE |
| rs11048591 | C | 26734991 | 0.336 | 0.021 | 0.004 | 1.20E-06 | 115060 | 2.05E-04 | 23.60 | FALSE |
| rs607087 | T | 109781909 | 0.292 | -0.022 | 0.005 | 1.60E-06 | 115060 | 2.00E-04 | 23.04 | FALSE |
| rs190366150 | T | 86270909 | 0.017 | -0.083 | 0.017 | 1.70E-06 | 115060 | 1.99E-04 | 22.94 | FALSE |
| rs11121396 | C | 9450899 | 0.413 | 0.020 | 0.004 | 1.80E-06 | 115060 | 1.98E-04 | 22.80 | FALSE |
| rs77849807 | G | 26152727 | 0.015 | 0.080 | 0.017 | 2.00E-06 | 115060 | 1.96E-04 | 22.55 | FALSE |
| rs631695 | G | 69283303 | 0.582 | 0.020 | 0.004 | 2.40E-06 | 115060 | 1.93E-04 | 22.21 | FALSE |
| rs116446940 | C | 103787181 | 0.009 | -0.108 | 0.023 | 2.60E-06 | 115060 | 1.92E-04 | 22.12 | FALSE |
| rs58087925 | T | 105983096 | 0.239 | -0.023 | 0.005 | 2.70E-06 | 115060 | 1.92E-04 | 22.05 | FALSE |
| rs1455590 | T | 106869973 | 0.233 | 0.023 | 0.005 | 3.00E-06 | 115060 | 1.89E-04 | 21.79 | FALSE |
| rs10518679 | C | 40382441 | 0.151 | 0.027 | 0.006 | 3.10E-06 | 115060 | 1.89E-04 | 21.77 | FALSE |
| rs3818871 | A | 51402055 | 0.277 | -0.022 | 0.005 | 3.10E-06 | 115060 | 1.89E-04 | 21.75 | FALSE |
| rs72623855 | A | 35445211 | 0.041 | -0.049 | 0.011 | 3.10E-06 | 115060 | 1.89E-04 | 21.75 | FALSE |
| rs56347111 | A | 221061116 | 0.234 | 0.023 | 0.005 | 3.20E-06 | 115060 | 1.88E-04 | 21.66 | FALSE |
| rs9274443 | G | 32634922 | 0.478 | -0.022 | 0.005 | 3.30E-06 | 115060 | 1.88E-04 | 21.61 | TRUE |
| rs819109 | A | 30227763 | 0.590 | 0.020 | 0.004 | 3.70E-06 | 115060 | 1.86E-04 | 21.41 | FALSE |
| rs10898 | T | 9536249 | 0.736 | 0.022 | 0.005 | 3.90E-06 | 115060 | 1.85E-04 | 21.33 | FALSE |
| rs12613605 | T | 43358910 | 0.213 | 0.023 | 0.005 | 4.10E-06 | 115060 | 1.84E-04 | 21.23 | FALSE |
| rs2112469 | A | 122933256 | 0.544 | 0.019 | 0.004 | 4.20E-06 | 115060 | 1.84E-04 | 21.17 | FALSE |
| rs150687233 | T | 23121847 | 0.354 | 0.020 | 0.004 | 4.60E-06 | 115060 | 1.83E-04 | 21.01 | FALSE |
| **Catalase** | | | | | | | | | | |
| **SNP** | **EA** | **Position** | **EAF** | **BETA** | **SE** | **P** | **N** | **R2** | **F** | **palindromic** |
| rs2745924 | T | 34399185 | 0.545 | 0.189 | 0.025 | 2.69E-14 | 3301 | 1.72E-02 | 57.82 | FALSE |
| rs700715 | C | 132726731 | 0.671 | -0.136 | 0.026 | 2.24E-07 | 3301 | 8.07E-03 | 26.85 | FALSE |
| rs56106813 | T | 103451593 | 0.067 | -0.257 | 0.051 | 5.01E-07 | 3301 | 7.60E-03 | 25.28 | FALSE |
| rs17089624 | C | 53358100 | 0.052 | -0.281 | 0.056 | 5.01E-07 | 3301 | 7.59E-03 | 25.22 | FALSE |
| rs4806509 | T | 54337096 | 0.672 | 0.143 | 0.029 | 6.76E-07 | 3301 | 7.40E-03 | 24.60 | FALSE |
| rs1685815 | A | 95605230 | 0.746 | -0.145 | 0.030 | 9.12E-07 | 3301 | 7.26E-03 | 24.11 | FALSE |
| rs116970161 | T | 100559970 | 0.018 | 0.472 | 0.097 | 1.12E-06 | 3301 | 7.13E-03 | 23.69 | FALSE |
| rs10918963 | C | 162213867 | 0.202 | -0.146 | 0.031 | 2.45E-06 | 3301 | 6.67E-03 | 22.14 | FALSE |
| rs73087640 | T | 238510855 | 0.097 | 0.195 | 0.041 | 2.57E-06 | 3301 | 6.66E-03 | 22.13 | FALSE |
| rs4970968 | T | 150612360 | 0.642 | 0.121 | 0.026 | 2.88E-06 | 3301 | 6.60E-03 | 21.91 | FALSE |
| rs13381678 | T | 43954046 | 0.132 | 0.171 | 0.037 | 2.88E-06 | 3301 | 6.58E-03 | 21.87 | FALSE |
| rs28549749 | C | 155308629 | 0.368 | 0.122 | 0.026 | 3.31E-06 | 3301 | 6.52E-03 | 21.66 | FALSE |
| rs116670019 | T | 55966573 | 0.018 | -0.497 | 0.107 | 3.55E-06 | 3301 | 6.47E-03 | 21.50 | FALSE |
| rs79696893 | G | 50135271 | 0.034 | 0.323 | 0.071 | 4.79E-06 | 3301 | 6.30E-03 | 20.91 | FALSE |
| **Gamma-tocopherol** | | | | | | | | | | |
| **SNP** | **EA** | **Position** | **EAF** | **BETA** | **SE** | **P** | **N** | **R2** | **F** | **palindromic** |
| rs1060467 | G | 16024538 | 0.410 | -0.023 | 0.005 | 2.61E-07 | 5822 | 4.58E-03 | 26.80 | FALSE |
| rs5994305 | G | 30820707 | 0.168 | -0.031 | 0.006 | 7.15E-07 | 5822 | 4.19E-03 | 24.51 | FALSE |
| rs261301 | C | 58686939 | 0.869 | -0.032 | 0.007 | 2.06E-06 | 5822 | 3.86E-03 | 22.55 | FALSE |
| rs7038957 | C | 106539439 | 0.169 | 0.029 | 0.006 | 3.86E-06 | 5822 | 3.67E-03 | 21.42 | FALSE |
| rs10077932 | T | 2450679 | 0.138 | -0.040 | 0.009 | 4.08E-06 | 5822 | 3.65E-03 | 21.34 | FALSE |
| rs1013104 | T | 12187236 | 0.435 | -0.021 | 0.005 | 3.83E-06 | 5822 | 3.62E-03 | 21.15 | FALSE |
| rs7350776 | G | 33689075 | 0.303 | -0.024 | 0.005 | 3.86E-06 | 5822 | 3.62E-03 | 21.12 | TRUE |
| **Glutathione peroxidase 7** | | | | | | | | | | |
| **SNP** | **EA** | **Position** | **EAF** | **BETA** | **SE** | **P** | **N** | **R2** | **F** | **palindromic** |
| rs1097234 | A | 53063559 | 0.175 | 0.563 | 0.031 | 1.62E-73 | 3301 | 9.04E-02 | 327.87 | FALSE |
| rs61821092 | G | 203052054 | 0.141 | -0.217 | 0.041 | 9.55E-08 | 3301 | 8.55E-03 | 28.44 | FALSE |
| rs76331340 | A | 9970464 | 0.049 | -0.317 | 0.060 | 1.38E-07 | 3301 | 8.35E-03 | 27.77 | FALSE |
| rs6993770 | T | 106581528 | 0.282 | -0.134 | 0.027 | 1.05E-06 | 3301 | 7.19E-03 | 23.90 | TRUE |
| rs7186574 | T | 26847795 | 0.151 | 0.171 | 0.036 | 1.51E-06 | 3301 | 6.95E-03 | 23.08 | FALSE |
| rs204523 | T | 44958980 | 0.811 | -0.150 | 0.032 | 1.95E-06 | 3301 | 6.82E-03 | 22.66 | TRUE |
| rs77041245 | A | 24995379 | 0.011 | -0.579 | 0.123 | 2.34E-06 | 3301 | 6.70E-03 | 22.25 | FALSE |
| rs12936913 | T | 77833916 | 0.282 | -0.127 | 0.027 | 3.09E-06 | 3301 | 6.56E-03 | 21.79 | FALSE |
| rs61797068 | C | 115902514 | 0.156 | 0.159 | 0.034 | 3.63E-06 | 3301 | 6.46E-03 | 21.45 | TRUE |
| rs12048380 | C | 62122956 | 0.854 | -0.173 | 0.037 | 3.72E-06 | 3301 | 6.44E-03 | 21.38 | FALSE |
| rs6892733 | A | 124599763 | 0.540 | 0.113 | 0.025 | 3.98E-06 | 3301 | 6.39E-03 | 21.20 | FALSE |
| rs73135263 | G | 55180294 | 0.020 | -0.423 | 0.092 | 4.17E-06 | 3301 | 6.38E-03 | 21.20 | TRUE |
| rs9883667 | A | 101806423 | 0.040 | -0.284 | 0.062 | 4.37E-06 | 3301 | 6.36E-03 | 21.11 | FALSE |
| rs116766476 | T | 90634753 | 0.012 | 0.553 | 0.121 | 4.79E-06 | 3301 | 6.31E-03 | 20.95 | FALSE |
| **Glutathione S-transferase A1** | | | | | | | | | | |
| **SNP** | **EA** | **Position** | **EAF** | **BETA** | **SE** | **P** | **N** | **R2** | **F** | **palindromic** |
| rs2290758 | A | 52662153 | 0.570 | 0.416 | 0.024 | 7.24E-69 | 3301 | 8.53E-02 | 307.47 | FALSE |
| rs79104405 | A | 2379204 | 0.024 | 0.446 | 0.082 | 4.68E-08 | 3301 | 8.95E-03 | 29.80 | FALSE |
| rs12214820 | A | 27537104 | 0.176 | 0.183 | 0.034 | 9.77E-08 | 3301 | 8.55E-03 | 28.46 | FALSE |
| rs2185401 | T | 203208365 | 0.032 | -0.350 | 0.071 | 8.13E-07 | 3301 | 7.31E-03 | 24.29 | FALSE |
| rs78409028 | A | 108978848 | 0.006 | 0.785 | 0.161 | 1.15E-06 | 3301 | 7.12E-03 | 23.66 | FALSE |
| rs117829315 | G | 98915741 | 0.013 | -0.616 | 0.127 | 1.32E-06 | 3301 | 7.03E-03 | 23.36 | FALSE |
| rs7819300 | T | 38480612 | 0.385 | -0.122 | 0.026 | 2.19E-06 | 3301 | 6.73E-03 | 22.35 | FALSE |
| rs60042638 | A | 243038707 | 0.104 | 0.212 | 0.045 | 2.40E-06 | 3301 | 6.68E-03 | 22.20 | FALSE |
| rs73073444 | T | 27241281 | 0.011 | -0.561 | 0.119 | 2.51E-06 | 3301 | 6.66E-03 | 22.13 | FALSE |
| rs75534686 | T | 29254672 | 0.023 | 0.406 | 0.087 | 3.02E-06 | 3301 | 6.56E-03 | 21.77 | FALSE |
| rs7720426 | A | 26065402 | 0.180 | -0.150 | 0.032 | 3.63E-06 | 3301 | 6.45E-03 | 21.42 | FALSE |
| **Hypoxanthine** | | | | | | | | | | |
| **SNP** | **EA** | **Position** | **EAF** | **BETA** | **SE** | **P** | **N** | **R2** | **F** | **palindromic** |
| rs16948294 | A | 94164895 | 0.069 | -0.041 | 0.009 | 1.35E-06 | 1745 | 1.32E-02 | 23.24 | TRUE |
| rs9379061 | G | 6735912 | 0.044 | 0.046 | 0.010 | 3.92E-06 | 1745 | 1.20E-02 | 21.19 | TRUE |
| rs6459467 | A | 16288192 | 0.389 | 0.020 | 0.003 | 1.93E-16 | 6941 | 9.32E-03 | 65.27 | FALSE |
| rs275721 | C | 40115920 | 0.491 | 0.016 | 0.003 | 9.64E-08 | 5196 | 5.44E-03 | 28.43 | FALSE |
| rs7644390 | A | 121990228 | 0.348 | 0.013 | 0.003 | 1.76E-07 | 6941 | 3.82E-03 | 26.62 | TRUE |
| rs2247330 | C | 96424122 | 0.348 | -0.012 | 0.003 | 4.56E-07 | 6941 | 3.53E-03 | 24.59 | FALSE |
| rs11260728 | C | 16658845 | 0.411 | -0.012 | 0.002 | 8.41E-07 | 6941 | 3.53E-03 | 24.58 | TRUE |
| rs11595079 | T | 87819082 | 0.095 | -0.021 | 0.005 | 1.89E-06 | 6941 | 3.25E-03 | 22.61 | FALSE |
| rs1824583 | G | 99064051 | 0.209 | 0.015 | 0.003 | 3.38E-06 | 6941 | 3.14E-03 | 21.87 | FALSE |
| rs2113305 | G | 79051997 | 0.735 | 0.013 | 0.003 | 4.95E-06 | 6941 | 3.07E-03 | 21.34 | FALSE |
| rs2145985 | T | 99174531 | 0.160 | -0.015 | 0.003 | 3.91E-06 | 6941 | 3.05E-03 | 21.21 | FALSE |
| rs4676313 | C | 107684447 | 0.122 | -0.018 | 0.004 | 3.54E-06 | 6941 | 3.03E-03 | 21.06 | FALSE |
| rs4776752 | A | 66451799 | 0.151 | 0.018 | 0.004 | 4.23E-06 | 6941 | 2.99E-03 | 20.83 | FALSE |
| **Kynurenine** | | | | | | | | | | |
| **SNP** | **EA** | **Position** | **EAF** | **BETA** | **SE** | **P** | **N** | **R2** | **F** | **palindromic** |
| rs16924894 | A | 24845525 | 0.024 | 0.081 | 0.015 | 2.33E-08 | 1764 | 1.73E-02 | 31.05 | TRUE |
| rs8051149 | A | 87878822 | 0.213 | 0.026 | 0.003 | 9.07E-26 | 7368 | 1.42E-02 | 106.47 | FALSE |
| rs3809198 | A | 96883306 | 0.020 | 0.055 | 0.012 | 4.28E-06 | 1764 | 1.19E-02 | 21.29 | FALSE |
| rs7548008 | G | 43202095 | 0.496 | -0.016 | 0.004 | 3.56E-06 | 1764 | 1.17E-02 | 20.87 | FALSE |
| rs6575634 | C | 98248954 | 0.382 | 0.016 | 0.004 | 4.84E-06 | 1764 | 1.14E-02 | 20.36 | FALSE |
| rs3184504 | C | 111884608 | 0.515 | -0.015 | 0.002 | 6.05E-18 | 7368 | 1.03E-02 | 76.80 | FALSE |
| rs10085935 | T | 39806267 | 0.378 | -0.010 | 0.002 | 3.33E-09 | 7368 | 4.96E-03 | 36.70 | FALSE |
| rs2491294 | G | 88302604 | 0.690 | 0.010 | 0.002 | 5.73E-07 | 5604 | 4.44E-03 | 24.99 | FALSE |
| rs21327 | G | 146528521 | 0.714 | -0.010 | 0.002 | 1.25E-07 | 7368 | 3.77E-03 | 27.85 | FALSE |
| rs10857319 | T | 159729794 | 0.348 | -0.009 | 0.002 | 9.45E-07 | 7368 | 3.46E-03 | 25.58 | FALSE |
| rs11646849 | A | 82817251 | 0.501 | -0.009 | 0.002 | 6.40E-07 | 7368 | 3.46E-03 | 25.58 | FALSE |
| rs1496635 | C | 31692461 | 0.659 | 0.009 | 0.002 | 2.12E-07 | 7368 | 3.46E-03 | 25.55 | FALSE |
| rs4820242 | A | 36982675 | 0.620 | -0.009 | 0.002 | 9.03E-07 | 7368 | 3.38E-03 | 24.99 | FALSE |
| rs6815057 | A | 97782257 | 0.579 | 0.009 | 0.002 | 1.12E-06 | 7368 | 3.38E-03 | 24.99 | FALSE |
| rs2375475 | T | 65211843 | 0.324 | -0.008 | 0.002 | 1.41E-06 | 7368 | 3.30E-03 | 24.41 | FALSE |
| rs3789978 | G | 32947193 | 0.153 | -0.012 | 0.003 | 1.32E-06 | 7368 | 3.27E-03 | 24.20 | FALSE |
| rs12082398 | C | 25234781 | 0.415 | -0.012 | 0.002 | 9.33E-07 | 7368 | 3.22E-03 | 23.76 | FALSE |
| rs16974854 | T | 68029032 | 0.038 | -0.023 | 0.005 | 1.15E-06 | 7368 | 3.22E-03 | 23.76 | FALSE |
| rs12937634 | C | 47444442 | 0.460 | 0.008 | 0.002 | 2.28E-06 | 7368 | 3.15E-03 | 23.26 | FALSE |
| rs1426134 | G | 14179729 | 0.629 | 0.008 | 0.002 | 3.84E-06 | 7368 | 3.07E-03 | 22.70 | TRUE |
| rs2320536 | T | 140801676 | 0.413 | 0.008 | 0.002 | 3.32E-06 | 7368 | 3.07E-03 | 22.70 | FALSE |
| rs2651516 | C | 53302309 | 0.342 | -0.008 | 0.002 | 4.30E-06 | 7368 | 3.00E-03 | 22.14 | FALSE |
| rs6770323 | T | 161850410 | 0.430 | -0.008 | 0.002 | 3.33E-06 | 7368 | 3.00E-03 | 22.14 | FALSE |
| rs9857268 | A | 83398721 | 0.411 | 0.008 | 0.002 | 3.07E-06 | 7368 | 3.00E-03 | 22.14 | FALSE |
| rs511797 | A | 95895139 | 0.126 | 0.012 | 0.003 | 3.77E-06 | 7368 | 2.88E-03 | 21.30 | FALSE |
| rs11593042 | A | 6933467 | 0.092 | 0.015 | 0.003 | 4.54E-06 | 7368 | 2.83E-03 | 20.93 | TRUE |
| **Kynurenine--oxoglutarate transaminase 3** | | | | | | | | | | |
| **SNP** | **EA** | **Position** | **EAF** | **BETA** | **SE** | **P** | **N** | **R2** | **F** | **palindromic** |
| rs9787133 | G | 89382664 | 0.494 | -0.172 | 0.025 | 2.88E-12 | 3301 | 1.45E-02 | 48.63 | TRUE |
| rs9384389 | C | 156209335 | 0.538 | -0.128 | 0.025 | 2.19E-07 | 3301 | 8.11E-03 | 26.96 | FALSE |
| rs59911114 | C | 120553574 | 0.017 | -0.515 | 0.102 | 4.07E-07 | 3301 | 7.71E-03 | 25.63 | FALSE |
| rs114543160 | A | 5223586 | 0.017 | -0.522 | 0.104 | 5.50E-07 | 3301 | 7.55E-03 | 25.08 | FALSE |
| rs117155162 | T | 73498184 | 0.009 | 0.716 | 0.145 | 7.41E-07 | 3301 | 7.36E-03 | 24.48 | FALSE |
| rs141274820 | G | 931075 | 0.009 | -0.666 | 0.138 | 1.29E-06 | 3301 | 7.05E-03 | 23.42 | FALSE |
| rs12935125 | T | 26948582 | 0.259 | 0.138 | 0.029 | 2.14E-06 | 3301 | 6.77E-03 | 22.48 | TRUE |
| rs66465679 | C | 55943930 | 0.174 | -0.162 | 0.034 | 2.34E-06 | 3301 | 6.73E-03 | 22.35 | FALSE |
| rs7500458 | G | 8690539 | 0.718 | 0.129 | 0.027 | 2.63E-06 | 3301 | 6.64E-03 | 22.05 | FALSE |
| rs1811 | G | 35434238 | 0.469 | 0.115 | 0.025 | 3.31E-06 | 3301 | 6.52E-03 | 21.65 | FALSE |
| rs150528455 | A | 26740555 | 0.020 | 0.468 | 0.102 | 4.07E-06 | 3301 | 6.39E-03 | 21.23 | FALSE |
| rs78857374 | T | 90168670 | 0.634 | -0.119 | 0.026 | 4.37E-06 | 3301 | 6.35E-03 | 21.10 | FALSE |
| rs200849103 | C | 134224776 | 0.128 | 0.175 | 0.038 | 4.79E-06 | 3301 | 6.30E-03 | 20.91 | FALSE |
| **Lactate** | | | | | | | | | | |
| **SNP** | **EA** | **Position** | **EAF** | **BETA** | **SE** | **P** | **N** | **R2** | **F** | **palindromic** |
| rs61835136 | C | 3139540 | 0.368 | 0.058 | 0.004 | 3.80E-39 | 114802 | 1.49E-03 | 171.33 | FALSE |
| rs7137828 | T | 111932800 | 0.516 | -0.042 | 0.004 | 6.80E-24 | 114802 | 8.84E-04 | 101.59 | FALSE |
| rs385893 | C | 4763176 | 0.523 | 0.039 | 0.004 | 2.30E-21 | 114802 | 7.84E-04 | 90.08 | FALSE |
| rs9389268 | G | 135419631 | 0.260 | 0.036 | 0.005 | 5.00E-14 | 114802 | 4.94E-04 | 56.71 | FALSE |
| rs11251686 | A | 3104881 | 0.778 | 0.039 | 0.005 | 1.40E-13 | 114802 | 4.77E-04 | 54.74 | FALSE |
| rs4665972 | C | 27598097 | 0.605 | -0.030 | 0.004 | 8.00E-13 | 114802 | 4.47E-04 | 51.28 | FALSE |
| rs6993770 | T | 106581528 | 0.288 | -0.030 | 0.005 | 6.20E-11 | 114802 | 3.72E-04 | 42.75 | TRUE |
| rs1473698 | T | 8629807 | 0.402 | -0.025 | 0.004 | 4.80E-09 | 114802 | 2.98E-04 | 34.25 | FALSE |
| rs12273368 | C | 119089742 | 0.272 | 0.027 | 0.005 | 6.40E-09 | 114802 | 2.93E-04 | 33.70 | FALSE |
| rs1909682 | G | 2966188 | 0.634 | 0.025 | 0.004 | 8.00E-09 | 114802 | 2.90E-04 | 33.28 | FALSE |
| rs390387 | G | 21920403 | 0.813 | 0.030 | 0.005 | 2.40E-08 | 114802 | 2.71E-04 | 31.16 | FALSE |
| rs59273177 | T | 201161291 | 0.020 | 0.081 | 0.015 | 4.40E-08 | 114802 | 2.61E-04 | 29.95 | TRUE |
| rs73193749 | T | 124371934 | 0.016 | -0.086 | 0.016 | 1.20E-07 | 114802 | 2.44E-04 | 28.03 | FALSE |
| rs10116352 | C | 4939466 | 0.900 | 0.035 | 0.007 | 3.70E-07 | 114802 | 2.25E-04 | 25.83 | TRUE |
| rs12865434 | G | 108970631 | 0.221 | -0.025 | 0.005 | 4.30E-07 | 114802 | 2.23E-04 | 25.57 | FALSE |
| rs73233340 | C | 125774106 | 0.029 | 0.063 | 0.012 | 4.60E-07 | 114802 | 2.22E-04 | 25.44 | FALSE |
| rs12376511 | C | 22142756 | 0.163 | -0.028 | 0.006 | 7.20E-07 | 114802 | 2.14E-04 | 24.55 | FALSE |
| rs2252214 | C | 297750 | 0.228 | -0.025 | 0.005 | 8.60E-07 | 114802 | 2.11E-04 | 24.22 | FALSE |
| rs34143161 | A | 8991813 | 0.129 | 0.031 | 0.006 | 8.60E-07 | 114802 | 2.11E-04 | 24.22 | FALSE |
| rs2692512 | A | 54022832 | 0.324 | -0.022 | 0.004 | 9.10E-07 | 114802 | 2.10E-04 | 24.10 | TRUE |
| rs56836735 | A | 184114611 | 0.204 | 0.025 | 0.005 | 1.10E-06 | 114802 | 2.06E-04 | 23.68 | TRUE |
| rs7889 | G | 31605448 | 0.641 | 0.021 | 0.004 | 1.50E-06 | 114802 | 2.02E-04 | 23.18 | TRUE |
| rs375072 | A | 142174972 | 0.044 | -0.048 | 0.010 | 1.80E-06 | 114802 | 1.98E-04 | 22.75 | TRUE |
| rs4323051 | A | 144998150 | 0.537 | -0.020 | 0.004 | 1.90E-06 | 114802 | 1.98E-04 | 22.73 | FALSE |
| rs34762299 | G | 40605099 | 0.188 | 0.025 | 0.005 | 2.00E-06 | 114802 | 1.97E-04 | 22.58 | FALSE |
| rs4790312 | C | 1981480 | 0.634 | -0.020 | 0.004 | 2.10E-06 | 114802 | 1.96E-04 | 22.53 | FALSE |
| rs10483169 | G | 33449418 | 0.017 | -0.080 | 0.017 | 2.10E-06 | 114802 | 1.96E-04 | 22.50 | FALSE |
| rs10139476 | T | 70403547 | 0.078 | 0.037 | 0.008 | 2.20E-06 | 114802 | 1.95E-04 | 22.38 | FALSE |
| rs72842819 | C | 7328821 | 0.121 | 0.030 | 0.006 | 2.60E-06 | 114802 | 1.92E-04 | 22.08 | FALSE |
| rs567131 | A | 229141494 | 0.735 | 0.022 | 0.005 | 2.70E-06 | 114802 | 1.92E-04 | 22.04 | FALSE |
| rs113902248 | G | 79699374 | 0.045 | -0.048 | 0.010 | 2.80E-06 | 114802 | 1.91E-04 | 21.97 | FALSE |
| rs1584926 | C | 94009292 | 0.046 | 0.046 | 0.010 | 2.90E-06 | 114802 | 1.90E-04 | 21.85 | FALSE |
| rs8086358 | G | 32393636 | 0.123 | 0.030 | 0.006 | 3.00E-06 | 114802 | 1.90E-04 | 21.83 | FALSE |
| rs56178803 | C | 13397699 | 0.076 | 0.037 | 0.008 | 3.60E-06 | 114802 | 1.87E-04 | 21.48 | FALSE |
| rs149720051 | C | 53278443 | 0.019 | -0.072 | 0.016 | 4.00E-06 | 114802 | 1.85E-04 | 21.25 | FALSE |
| rs738800 | A | 24158315 | 0.824 | 0.025 | 0.005 | 4.30E-06 | 114802 | 1.84E-04 | 21.11 | FALSE |
| **Monounsaturated fatty acids** | | | | | | | | | | |
| **SNP** | **EA** | **Position** | **EAF** | **BETA** | **SE** | **P** | **N** | **R2** | **F** | **palindromic** |
| rs964184 | C | 116648917 | 0.867 | -0.205 | 0.006 | 1.00E-200 | 114999 | 9.95E-03 | 1155.74 | TRUE |
| rs1260326 | C | 27730940 | 0.604 | -0.111 | 0.004 | 1.70E-158 | 114999 | 6.06E-03 | 700.94 | FALSE |
| rs1002687 | A | 62963737 | 0.645 | 0.091 | 0.004 | 6.40E-105 | 114999 | 3.94E-03 | 455.25 | FALSE |
| rs28601761 | G | 126500031 | 0.420 | -0.089 | 0.004 | 3.30E-104 | 114999 | 3.94E-03 | 454.35 | TRUE |
| rs328 | G | 19819724 | 0.100 | -0.143 | 0.007 | 1.10E-101 | 114999 | 3.84E-03 | 443.79 | TRUE |
| rs10455872 | G | 161010118 | 0.079 | -0.149 | 0.008 | 9.20E-89 | 114999 | 3.35E-03 | 386.37 | FALSE |
| rs3812316 | G | 73020337 | 0.129 | -0.113 | 0.006 | 1.30E-76 | 114999 | 3.01E-03 | 347.36 | TRUE |
| rs5112 | G | 45430280 | 0.534 | 0.079 | 0.004 | 1.80E-75 | 114999 | 2.83E-03 | 326.08 | TRUE |
| rs117733303 | G | 160922870 | 0.019 | -0.263 | 0.015 | 1.20E-67 | 114999 | 2.61E-03 | 300.51 | FALSE |
| rs261290 | C | 58678720 | 0.655 | -0.074 | 0.004 | 9.00E-67 | 114999 | 2.54E-03 | 293.00 | FALSE |
| rs58542926 | T | 19379549 | 0.074 | -0.118 | 0.008 | 9.90E-53 | 114999 | 1.98E-03 | 227.61 | FALSE |
| rs174564 | G | 61588305 | 0.347 | 0.064 | 0.004 | 3.30E-52 | 114999 | 1.96E-03 | 225.86 | FALSE |
| rs4564803 | T | 21205502 | 0.228 | -0.072 | 0.005 | 5.10E-50 | 114999 | 1.89E-03 | 217.57 | FALSE |
| rs633695 | G | 58725839 | 0.292 | 0.056 | 0.005 | 2.10E-35 | 114999 | 1.33E-03 | 152.85 | FALSE |
| rs141469619 | G | 116714293 | 0.010 | 0.242 | 0.022 | 5.40E-32 | 114999 | 1.09E-03 | 125.63 | FALSE |
| rs116843064 | A | 8429323 | 0.020 | -0.163 | 0.015 | 3.80E-29 | 114999 | 1.07E-03 | 123.65 | FALSE |
| rs9271573 | C | 32590501 | 0.594 | 0.044 | 0.004 | 6.00E-25 | 114999 | 9.51E-04 | 109.50 | FALSE |
| rs4704834 | G | 156443066 | 0.644 | 0.044 | 0.004 | 2.20E-26 | 114999 | 9.35E-04 | 107.68 | FALSE |
| rs139974673 | C | 44027885 | 0.026 | 0.121 | 0.013 | 1.70E-21 | 114999 | 7.66E-04 | 88.13 | FALSE |
| rs13108218 | G | 3443931 | 0.615 | -0.038 | 0.004 | 6.10E-20 | 114999 | 6.88E-04 | 79.19 | FALSE |
| rs149615216 | T | 47106028 | 0.011 | 0.169 | 0.020 | 2.20E-18 | 114999 | 6.28E-04 | 72.31 | FALSE |
| rs1128249 | T | 165528624 | 0.392 | -0.035 | 0.004 | 6.30E-18 | 114999 | 6.18E-04 | 71.11 | FALSE |
| rs3936511 | G | 55860781 | 0.192 | 0.042 | 0.005 | 8.40E-16 | 114999 | 5.79E-04 | 66.68 | FALSE |
| rs10102524 | G | 11534907 | 0.549 | -0.033 | 0.004 | 2.00E-15 | 114999 | 5.62E-04 | 64.71 | FALSE |
| rs632057 | G | 139834012 | 0.628 | -0.032 | 0.004 | 3.10E-13 | 114999 | 4.86E-04 | 55.92 | FALSE |
| rs10761716 | G | 64882300 | 0.441 | -0.030 | 0.004 | 5.40E-14 | 114999 | 4.74E-04 | 54.54 | TRUE |
| rs4846914 | A | 230295691 | 0.605 | -0.030 | 0.004 | 1.60E-13 | 114999 | 4.55E-04 | 52.32 | FALSE |
| rs1471251 | T | 87976359 | 0.397 | 0.030 | 0.004 | 1.70E-13 | 114999 | 4.48E-04 | 51.54 | TRUE |
| rs182611493 | G | 19458388 | 0.013 | -0.141 | 0.020 | 9.20E-14 | 114999 | 4.45E-04 | 51.18 | FALSE |
| rs28752924 | C | 31303922 | 0.446 | 0.030 | 0.004 | 7.20E-09 | 114999 | 4.38E-04 | 50.36 | FALSE |
| rs28752924 | C | 31303922 | 0.446 | 0.030 | 0.004 | 7.20E-09 | 114999 | 4.38E-04 | 50.36 | FALSE |
| rs7679 | C | 44576502 | 0.186 | 0.037 | 0.005 | 1.70E-13 | 114999 | 4.24E-04 | 48.77 | FALSE |
| rs1052248 | A | 31556581 | 0.258 | 0.032 | 0.005 | 4.60E-10 | 114999 | 4.01E-04 | 46.12 | TRUE |
| rs1394092 | T | 136196071 | 0.727 | 0.031 | 0.005 | 1.80E-12 | 114999 | 3.99E-04 | 45.87 | FALSE |
| rs1540037 | G | 47182664 | 0.778 | 0.034 | 0.005 | 5.50E-11 | 114999 | 3.98E-04 | 45.73 | FALSE |
| rs7140110 | C | 114544024 | 0.299 | 0.030 | 0.004 | 5.70E-12 | 114999 | 3.95E-04 | 45.47 | FALSE |
| rs739320 | C | 49261368 | 0.607 | -0.028 | 0.004 | 2.70E-10 | 114999 | 3.84E-04 | 44.16 | FALSE |
| rs146203232 | T | 160543148 | 0.075 | 0.052 | 0.008 | 4.90E-12 | 114999 | 3.82E-04 | 43.96 | FALSE |
| rs3198697 | T | 15129940 | 0.407 | -0.027 | 0.004 | 2.80E-12 | 114999 | 3.73E-04 | 42.89 | FALSE |
| rs72555385 | G | 73123473 | 0.049 | 0.061 | 0.009 | 1.40E-10 | 114999 | 3.59E-04 | 41.34 | FALSE |
| rs60960031 | A | 21530659 | 0.403 | -0.026 | 0.004 | 2.90E-10 | 114999 | 3.40E-04 | 39.15 | FALSE |
| rs534417 | G | 23784965 | 0.875 | 0.038 | 0.006 | 2.20E-10 | 114999 | 3.37E-04 | 38.75 | FALSE |
| rs11895352 | T | 20367135 | 0.475 | -0.025 | 0.004 | 2.50E-10 | 114999 | 3.30E-04 | 37.94 | FALSE |
| rs142385484 | T | 50016759 | 0.147 | -0.036 | 0.006 | 1.60E-10 | 114999 | 3.30E-04 | 37.92 | FALSE |
| rs2740488 | C | 107661742 | 0.265 | -0.028 | 0.005 | 2.30E-09 | 114999 | 3.27E-04 | 37.64 | FALSE |
| rs6606725 | A | 109905368 | 0.440 | 0.025 | 0.004 | 1.30E-10 | 114999 | 3.22E-04 | 37.06 | FALSE |
| rs2478236 | A | 94797973 | 0.404 | -0.025 | 0.004 | 3.00E-11 | 114999 | 3.21E-04 | 36.95 | FALSE |
| rs11854318 | A | 58571982 | 0.275 | -0.028 | 0.005 | 3.30E-09 | 114999 | 3.19E-04 | 36.74 | FALSE |
| rs11940694 | G | 39414993 | 0.605 | 0.025 | 0.004 | 6.70E-10 | 114999 | 3.17E-04 | 36.41 | FALSE |
| rs2081687 | C | 59388565 | 0.663 | -0.026 | 0.004 | 1.10E-09 | 114999 | 3.12E-04 | 35.84 | FALSE |
| rs2943635 | T | 227077377 | 0.680 | 0.026 | 0.004 | 6.30E-09 | 114999 | 3.08E-04 | 35.46 | FALSE |
| rs2000999 | A | 72108093 | 0.189 | 0.030 | 0.005 | 4.50E-09 | 114999 | 2.90E-04 | 33.38 | FALSE |
| rs12601919 | G | 65826861 | 0.189 | 0.030 | 0.005 | 1.10E-08 | 114999 | 2.84E-04 | 32.67 | FALSE |
| rs7979473 | G | 121420260 | 0.613 | -0.024 | 0.004 | 2.90E-08 | 114999 | 2.79E-04 | 32.11 | FALSE |
| rs6938550 | A | 20462138 | 0.914 | -0.041 | 0.007 | 2.00E-08 | 114999 | 2.75E-04 | 31.61 | FALSE |
| rs12916 | C | 74656539 | 0.400 | 0.023 | 0.004 | 1.90E-08 | 114999 | 2.72E-04 | 31.24 | FALSE |
| rs602633 | G | 109821511 | 0.783 | 0.028 | 0.005 | 5.30E-09 | 114999 | 2.71E-04 | 31.12 | FALSE |
| rs67981690 | G | 21343886 | 0.129 | 0.034 | 0.006 | 8.10E-09 | 114999 | 2.69E-04 | 30.96 | FALSE |
| rs2721961 | G | 116657911 | 0.281 | -0.025 | 0.005 | 6.40E-08 | 114999 | 2.69E-04 | 30.90 | FALSE |
| rs76895963 | G | 4384844 | 0.021 | -0.088 | 0.016 | 3.20E-08 | 114999 | 2.69E-04 | 30.89 | FALSE |
| rs2035816 | G | 100508556 | 0.084 | -0.041 | 0.007 | 1.30E-08 | 114999 | 2.68E-04 | 30.84 | FALSE |
| rs59347135 | G | 19750044 | 0.046 | 0.055 | 0.010 | 1.60E-08 | 114999 | 2.65E-04 | 30.44 | TRUE |
| rs2070971 | T | 44197583 | 0.137 | 0.033 | 0.006 | 7.30E-08 | 114999 | 2.63E-04 | 30.23 | FALSE |
| rs112259268 | A | 41874745 | 0.029 | 0.068 | 0.012 | 6.60E-09 | 114999 | 2.62E-04 | 30.16 | FALSE |
| rs62222988 | C | 40569508 | 0.374 | 0.023 | 0.004 | 7.10E-09 | 114999 | 2.57E-04 | 29.61 | FALSE |
| rs998584 | A | 43757896 | 0.482 | 0.022 | 0.004 | 2.00E-08 | 114999 | 2.56E-04 | 29.39 | FALSE |
| rs9910747 | C | 17433959 | 0.062 | 0.046 | 0.008 | 8.50E-08 | 114999 | 2.54E-04 | 29.22 | FALSE |
| rs117188729 | A | 8415076 | 0.031 | -0.064 | 0.012 | 5.50E-08 | 114999 | 2.53E-04 | 29.10 | TRUE |
| rs11172134 | A | 57645789 | 0.201 | -0.027 | 0.005 | 1.10E-07 | 114999 | 2.51E-04 | 28.89 | TRUE |
| rs2513048 | C | 62183281 | 0.712 | -0.024 | 0.005 | 1.60E-07 | 114999 | 2.43E-04 | 27.92 | FALSE |
| rs12055389 | T | 161091952 | 0.054 | 0.048 | 0.009 | 7.40E-08 | 114999 | 2.42E-04 | 27.89 | FALSE |
| rs1890896 | C | 93641757 | 0.527 | -0.022 | 0.004 | 3.70E-08 | 114999 | 2.40E-04 | 27.66 | FALSE |
| rs641154 | T | 234864214 | 0.493 | 0.021 | 0.004 | 5.10E-08 | 114999 | 2.40E-04 | 27.56 | FALSE |
| rs10472290 | T | 37772566 | 0.199 | -0.027 | 0.005 | 3.50E-07 | 114999 | 2.38E-04 | 27.43 | FALSE |
| rs11635458 | C | 65599531 | 0.875 | 0.032 | 0.006 | 1.10E-07 | 114999 | 2.37E-04 | 27.30 | FALSE |
| rs9616847 | T | 50868669 | 0.388 | 0.022 | 0.004 | 2.90E-07 | 114999 | 2.37E-04 | 27.23 | TRUE |
| rs4491981 | A | 171563776 | 0.241 | -0.025 | 0.005 | 7.40E-08 | 114999 | 2.35E-04 | 27.02 | FALSE |
| rs7402939 | C | 99183876 | 0.624 | 0.022 | 0.004 | 1.30E-07 | 114999 | 2.34E-04 | 26.91 | FALSE |
| rs11776943 | T | 18280550 | 0.075 | 0.039 | 0.008 | 2.30E-07 | 114999 | 2.20E-04 | 25.28 | FALSE |
| rs35764948 | A | 43213707 | 0.055 | 0.045 | 0.009 | 3.70E-07 | 114999 | 2.19E-04 | 25.19 | TRUE |
| rs1018070 | A | 18273627 | 0.054 | 0.045 | 0.009 | 1.20E-07 | 114999 | 2.18E-04 | 25.13 | TRUE |
| rs13188623 | C | 67492589 | 0.183 | -0.027 | 0.005 | 2.40E-07 | 114999 | 2.18E-04 | 25.08 | FALSE |
| rs3860847 | A | 126631799 | 0.213 | 0.025 | 0.005 | 8.30E-07 | 114999 | 2.17E-04 | 24.98 | FALSE |
| rs6102034 | G | 39186590 | 0.323 | -0.022 | 0.004 | 1.50E-07 | 114999 | 2.13E-04 | 24.53 | TRUE |
| rs5020545 | T | 77414988 | 0.447 | 0.020 | 0.004 | 1.20E-07 | 114999 | 2.09E-04 | 24.00 | FALSE |
| rs7038652 | A | 138158957 | 0.065 | -0.042 | 0.009 | 1.20E-06 | 114999 | 2.08E-04 | 23.87 | FALSE |
| rs3829125 | G | 5247784 | 0.154 | -0.028 | 0.006 | 6.30E-07 | 114999 | 2.07E-04 | 23.76 | TRUE |
| rs2849049 | A | 15301428 | 0.327 | 0.021 | 0.004 | 1.60E-06 | 114999 | 2.06E-04 | 23.73 | FALSE |
| rs16895971 | C | 17884986 | 0.137 | 0.029 | 0.006 | 2.10E-06 | 114999 | 2.06E-04 | 23.65 | FALSE |
| rs7323466 | C | 100671869 | 0.651 | -0.021 | 0.004 | 3.30E-06 | 114999 | 2.05E-04 | 23.63 | FALSE |
| rs2915400 | C | 14364951 | 0.768 | 0.024 | 0.005 | 7.30E-07 | 114999 | 2.05E-04 | 23.61 | FALSE |
| rs73022312 | G | 160837528 | 0.011 | 0.097 | 0.020 | 2.30E-06 | 114999 | 2.03E-04 | 23.41 | FALSE |
| rs72784652 | T | 90365475 | 0.061 | -0.041 | 0.009 | 1.50E-06 | 114999 | 2.02E-04 | 23.24 | FALSE |
| rs511346 | C | 99706324 | 0.437 | 0.020 | 0.004 | 3.80E-07 | 114999 | 2.01E-04 | 23.16 | FALSE |
| rs7704383 | T | 78602472 | 0.569 | 0.020 | 0.004 | 1.70E-06 | 114999 | 2.01E-04 | 23.07 | FALSE |
| rs10884966 | A | 112185596 | 0.346 | -0.021 | 0.004 | 4.00E-07 | 114999 | 2.00E-04 | 23.00 | FALSE |
| rs78096412 | G | 218227288 | 0.110 | -0.032 | 0.007 | 1.90E-06 | 114999 | 1.99E-04 | 22.90 | FALSE |
| rs2889 | G | 22875909 | 0.315 | -0.021 | 0.004 | 1.20E-06 | 114999 | 1.96E-04 | 22.55 | FALSE |
| rs141250728 | C | 52321809 | 0.014 | -0.083 | 0.017 | 2.60E-06 | 114999 | 1.95E-04 | 22.44 | FALSE |
| rs4541525 | C | 129090020 | 0.306 | -0.023 | 0.005 | 2.90E-06 | 114999 | 1.95E-04 | 22.43 | FALSE |
| rs224424 | G | 34147998 | 0.212 | -0.024 | 0.005 | 1.20E-06 | 114999 | 1.95E-04 | 22.41 | FALSE |
| rs2800709 | G | 127439297 | 0.520 | 0.019 | 0.004 | 1.10E-06 | 114999 | 1.93E-04 | 22.22 | FALSE |
| rs10954732 | A | 75611149 | 0.673 | -0.021 | 0.004 | 2.00E-06 | 114999 | 1.93E-04 | 22.21 | FALSE |
| rs150350500 | C | 49625933 | 0.013 | -0.087 | 0.019 | 2.00E-06 | 114999 | 1.93E-04 | 22.17 | FALSE |
| rs79287178 | A | 172294500 | 0.031 | 0.058 | 0.012 | 1.70E-06 | 114999 | 1.92E-04 | 22.07 | FALSE |
| rs138345261 | A | 73708234 | 0.024 | 0.064 | 0.014 | 1.50E-06 | 114999 | 1.91E-04 | 21.98 | FALSE |
| rs184089815 | A | 160093887 | 0.016 | -0.075 | 0.016 | 1.40E-06 | 114999 | 1.87E-04 | 21.56 | FALSE |
| rs6715200 | T | 169919751 | 0.450 | 0.019 | 0.004 | 1.50E-06 | 114999 | 1.87E-04 | 21.53 | TRUE |
| rs4683708 | T | 142653145 | 0.533 | -0.019 | 0.004 | 1.60E-06 | 114999 | 1.86E-04 | 21.44 | FALSE |
| rs213479 | T | 54861827 | 0.469 | -0.019 | 0.004 | 3.30E-06 | 114999 | 1.86E-04 | 21.42 | FALSE |
| rs77566028 | C | 110191606 | 0.015 | 0.078 | 0.017 | 4.90E-06 | 114999 | 1.86E-04 | 21.42 | FALSE |
| rs4976647 | C | 176788622 | 0.334 | 0.020 | 0.004 | 1.70E-06 | 114999 | 1.84E-04 | 21.18 | FALSE |
| rs78669871 | T | 39713052 | 0.190 | 0.024 | 0.005 | 4.40E-07 | 114999 | 1.84E-04 | 21.18 | TRUE |
| rs11040351 | A | 49390971 | 0.427 | -0.019 | 0.004 | 3.50E-06 | 114999 | 1.84E-04 | 21.17 | FALSE |
| rs633389 | T | 116667337 | 0.016 | 0.076 | 0.017 | 2.10E-06 | 114999 | 1.84E-04 | 21.14 | FALSE |
| rs6689335 | C | 219628682 | 0.418 | 0.019 | 0.004 | 3.90E-06 | 114999 | 1.82E-04 | 20.94 | FALSE |
| rs11076176 | G | 57007446 | 0.168 | -0.026 | 0.006 | 3.50E-06 | 114999 | 1.82E-04 | 20.89 | FALSE |
| rs115847023 | C | 185209882 | 0.007 | -0.110 | 0.024 | 1.40E-06 | 114999 | 1.80E-04 | 20.65 | FALSE |
| rs1615528 | C | 62527825 | 0.258 | -0.021 | 0.005 | 1.30E-06 | 114999 | 1.79E-04 | 20.64 | FALSE |
| rs195445 | T | 61744342 | 0.688 | 0.020 | 0.004 | 2.80E-06 | 114999 | 1.79E-04 | 20.59 | FALSE |
| rs7979454 | C | 11796909 | 0.473 | 0.019 | 0.004 | 1.50E-06 | 114999 | 1.79E-04 | 20.56 | TRUE |
| rs112495979 | A | 98779896 | 0.166 | -0.025 | 0.006 | 3.40E-06 | 114999 | 1.76E-04 | 20.27 | FALSE |
| rs62183700 | C | 203740157 | 0.329 | 0.020 | 0.004 | 7.70E-07 | 114999 | 1.76E-04 | 20.21 | TRUE |
| rs10928470 | G | 134689936 | 0.771 | 0.022 | 0.005 | 4.80E-06 | 114999 | 1.73E-04 | 19.86 | FALSE |
| rs8185771 | T | 43651106 | 0.956 | 0.045 | 0.010 | 1.80E-06 | 114999 | 1.72E-04 | 19.80 | FALSE |
| rs1546224 | T | 63696548 | 0.309 | 0.020 | 0.004 | 4.40E-06 | 114999 | 1.72E-04 | 19.74 | FALSE |
| rs11620783 | T | 24871530 | 0.432 | 0.018 | 0.004 | 2.80E-06 | 114999 | 1.71E-04 | 19.69 | FALSE |
| rs113993396 | G | 112117304 | 0.086 | 0.039 | 0.009 | 4.20E-06 | 114999 | 1.71E-04 | 19.68 | TRUE |
| rs55703318 | T | 118604486 | 0.081 | 0.034 | 0.008 | 4.50E-06 | 114999 | 1.70E-04 | 19.56 | FALSE |
| rs9327640 | G | 132386105 | 0.511 | -0.018 | 0.004 | 2.00E-06 | 114999 | 1.70E-04 | 19.51 | FALSE |
| rs6973520 | T | 128740443 | 0.506 | -0.018 | 0.004 | 2.50E-06 | 114999 | 1.67E-04 | 19.19 | FALSE |
| **Polyunsaturated fatty acids** | | | | | | | | | | |
| **SNP** | **EA** | **Position** | **EAF** | **BETA** | **SE** | **P** | **N** | **R2** | **F** | **palindromic** |
| rs261290 | C | 58678720 | 0.655 | -0.112 | 0.004 | 1.20E-157 | 114999 | 6.05E-03 | 700.01 | FALSE |
| rs964184 | C | 116648917 | 0.867 | -0.150 | 0.006 | 5.20E-146 | 114999 | 5.53E-03 | 639.98 | TRUE |
| rs102275 | C | 61557803 | 0.350 | -0.097 | 0.004 | 3.20E-125 | 114999 | 4.63E-03 | 534.97 | FALSE |
| rs1002687 | A | 62963737 | 0.645 | 0.096 | 0.004 | 2.00E-120 | 114999 | 4.51E-03 | 520.60 | FALSE |
| rs58542926 | T | 19379549 | 0.074 | -0.152 | 0.008 | 1.50E-91 | 114999 | 3.41E-03 | 393.70 | FALSE |
| rs633695 | G | 58725839 | 0.292 | 0.084 | 0.004 | 1.10E-79 | 114999 | 3.09E-03 | 355.89 | FALSE |
| rs1260326 | C | 27730940 | 0.604 | -0.076 | 0.004 | 1.60E-77 | 114999 | 2.98E-03 | 343.27 | FALSE |
| rs112875651 | A | 126506694 | 0.392 | -0.077 | 0.004 | 4.60E-78 | 114999 | 2.96E-03 | 341.25 | FALSE |
| rs77960347 | G | 47109955 | 0.013 | 0.277 | 0.018 | 5.70E-58 | 114999 | 2.15E-03 | 247.75 | FALSE |
| rs142158911 | A | 11190534 | 0.117 | -0.092 | 0.006 | 6.70E-50 | 114999 | 1.86E-03 | 213.72 | FALSE |
| rs9304381 | T | 47158234 | 0.818 | 0.074 | 0.005 | 3.80E-46 | 114999 | 1.73E-03 | 199.16 | FALSE |
| rs183130 | T | 56991363 | 0.324 | 0.056 | 0.004 | 7.20E-41 | 114999 | 1.47E-03 | 168.93 | FALSE |
| rs672889 | G | 21319016 | 0.860 | 0.075 | 0.006 | 7.30E-41 | 114999 | 1.45E-03 | 166.78 | FALSE |
| rs1461729 | G | 9187242 | 0.899 | 0.086 | 0.007 | 1.30E-38 | 114999 | 1.43E-03 | 164.23 | FALSE |
| rs9295128 | T | 160751531 | 0.017 | -0.203 | 0.016 | 2.90E-39 | 114999 | 1.41E-03 | 162.50 | FALSE |
| rs34121855 | G | 73040814 | 0.204 | -0.061 | 0.005 | 5.30E-34 | 114999 | 1.30E-03 | 149.24 | FALSE |
| rs12740374 | T | 109817590 | 0.221 | -0.059 | 0.005 | 3.30E-34 | 114999 | 1.29E-03 | 147.97 | FALSE |
| rs6882345 | A | 156397673 | 0.633 | 0.046 | 0.004 | 3.60E-29 | 114999 | 1.04E-03 | 119.85 | FALSE |
| rs2740488 | C | 107661742 | 0.265 | -0.049 | 0.005 | 7.20E-27 | 114999 | 1.00E-03 | 115.54 | FALSE |
| rs3843482 | G | 74639259 | 0.374 | 0.042 | 0.004 | 2.70E-25 | 114999 | 9.05E-04 | 104.16 | FALSE |
| rs28383314 | C | 32587213 | 0.623 | 0.040 | 0.004 | 1.70E-19 | 114999 | 8.22E-04 | 94.57 | FALSE |
| rs6938647 | C | 160986915 | 0.782 | -0.048 | 0.005 | 3.30E-23 | 114999 | 8.03E-04 | 92.41 | FALSE |
| rs1081105 | C | 45412955 | 0.028 | 0.118 | 0.012 | 1.40E-22 | 114999 | 7.94E-04 | 91.41 | FALSE |
| rs56322906 | A | 11346155 | 0.035 | -0.104 | 0.011 | 2.20E-21 | 114999 | 7.91E-04 | 91.04 | FALSE |
| rs11854242 | T | 58571910 | 0.275 | -0.043 | 0.005 | 4.50E-22 | 114999 | 7.81E-04 | 89.93 | FALSE |
| rs72997616 | A | 75474195 | 0.094 | -0.065 | 0.007 | 8.40E-21 | 114999 | 7.58E-04 | 87.19 | FALSE |
| rs6547409 | T | 21190209 | 0.051 | -0.085 | 0.009 | 4.30E-22 | 114999 | 7.30E-04 | 84.00 | FALSE |
| rs79429216 | A | 45445517 | 0.013 | 0.162 | 0.018 | 2.50E-20 | 114999 | 7.06E-04 | 81.21 | FALSE |
| rs13108218 | G | 3443931 | 0.615 | -0.034 | 0.004 | 1.60E-17 | 114999 | 5.88E-04 | 67.68 | FALSE |
| rs870526 | T | 20369562 | 0.521 | -0.032 | 0.004 | 2.80E-16 | 114999 | 5.54E-04 | 63.69 | FALSE |
| rs1883711 | C | 39179822 | 0.031 | 0.093 | 0.012 | 9.50E-17 | 114999 | 5.40E-04 | 62.08 | TRUE |
| rs7970695 | A | 121423376 | 0.621 | -0.033 | 0.004 | 2.00E-15 | 114999 | 5.31E-04 | 61.14 | FALSE |
| rs496654 | C | 234851165 | 0.517 | 0.030 | 0.004 | 5.60E-15 | 114999 | 4.76E-04 | 54.72 | FALSE |
| rs11789603 | T | 107647019 | 0.109 | 0.048 | 0.006 | 7.60E-14 | 114999 | 4.71E-04 | 54.15 | FALSE |
| rs4299376 | T | 44072576 | 0.676 | -0.032 | 0.004 | 7.30E-14 | 114999 | 4.64E-04 | 53.35 | FALSE |
| rs34955778 | C | 15139594 | 0.420 | -0.029 | 0.004 | 1.30E-12 | 114999 | 4.49E-04 | 51.64 | FALSE |
| rs4860948 | A | 69340991 | 0.244 | 0.034 | 0.005 | 4.90E-13 | 114999 | 4.48E-04 | 51.57 | TRUE |
| rs115478735 | T | 136149711 | 0.183 | 0.037 | 0.005 | 7.40E-14 | 114999 | 4.47E-04 | 51.40 | TRUE |
| rs114863007 | A | 34729158 | 0.095 | -0.048 | 0.007 | 4.10E-13 | 114999 | 4.19E-04 | 48.22 | FALSE |
| rs2326077 | T | 59385919 | 0.663 | -0.028 | 0.004 | 1.60E-11 | 114999 | 3.86E-04 | 44.42 | FALSE |
| rs6602911 | T | 114547372 | 0.360 | 0.028 | 0.004 | 5.80E-11 | 114999 | 3.85E-04 | 44.25 | FALSE |
| rs4766578 | A | 111904371 | 0.503 | 0.027 | 0.004 | 8.10E-12 | 114999 | 3.84E-04 | 44.20 | TRUE |
| rs4008004 | A | 15306292 | 0.222 | 0.032 | 0.005 | 3.80E-11 | 114999 | 3.77E-04 | 43.34 | FALSE |
| rs534417 | G | 23784965 | 0.875 | 0.039 | 0.006 | 1.10E-10 | 114999 | 3.52E-04 | 40.44 | FALSE |
| rs2737245 | T | 116658583 | 0.279 | -0.029 | 0.005 | 2.00E-10 | 114999 | 3.49E-04 | 40.11 | FALSE |
| rs75406471 | A | 5257647 | 0.155 | -0.035 | 0.006 | 4.20E-10 | 114999 | 3.46E-04 | 39.80 | FALSE |
| rs112866833 | T | 46200690 | 0.290 | 0.028 | 0.004 | 1.40E-10 | 114999 | 3.37E-04 | 38.82 | FALSE |
| rs115413710 | C | 31180819 | 0.044 | 0.060 | 0.010 | 8.10E-12 | 114999 | 3.30E-04 | 38.00 | FALSE |
| rs1800961 | T | 43042364 | 0.030 | -0.072 | 0.012 | 1.60E-09 | 114999 | 3.23E-04 | 37.15 | FALSE |
| rs4561509 | A | 45781799 | 0.502 | 0.024 | 0.004 | 7.60E-10 | 114999 | 3.05E-04 | 35.03 | FALSE |
| rs3770586 | T | 169828995 | 0.484 | -0.024 | 0.004 | 1.00E-09 | 114999 | 3.03E-04 | 34.88 | FALSE |
| rs141469619 | G | 116714293 | 0.010 | 0.125 | 0.021 | 1.30E-10 | 114999 | 3.01E-04 | 34.59 | FALSE |
| rs12363232 | T | 47648042 | 0.350 | -0.024 | 0.004 | 1.90E-09 | 114999 | 2.87E-04 | 33.07 | FALSE |
| rs3822855 | T | 116316882 | 0.401 | 0.024 | 0.004 | 6.40E-09 | 114999 | 2.86E-04 | 32.92 | FALSE |
| rs4986970 | T | 67976320 | 0.034 | -0.064 | 0.011 | 1.10E-08 | 114999 | 2.84E-04 | 32.68 | TRUE |
| rs9616847 | T | 50868669 | 0.388 | 0.023 | 0.004 | 3.50E-08 | 114999 | 2.77E-04 | 31.82 | TRUE |
| rs2378390 | A | 34154937 | 0.141 | -0.032 | 0.006 | 1.20E-08 | 114999 | 2.70E-04 | 31.11 | FALSE |
| rs10096633 | T | 19830921 | 0.124 | -0.034 | 0.006 | 3.10E-08 | 114999 | 2.69E-04 | 30.89 | FALSE |
| rs5754102 | A | 21927231 | 0.183 | -0.029 | 0.005 | 2.30E-08 | 114999 | 2.60E-04 | 29.94 | FALSE |
| rs10419198 | T | 50038017 | 0.253 | 0.025 | 0.005 | 4.40E-08 | 114999 | 2.54E-04 | 29.17 | FALSE |
| rs78277979 | A | 47276942 | 0.142 | 0.031 | 0.006 | 1.40E-07 | 114999 | 2.51E-04 | 28.82 | FALSE |
| rs12970 | A | 117074109 | 0.061 | -0.045 | 0.008 | 6.80E-08 | 114999 | 2.49E-04 | 28.63 | FALSE |
| rs6750775 | C | 165452415 | 0.082 | -0.039 | 0.007 | 3.70E-07 | 114999 | 2.43E-04 | 27.95 | TRUE |
| rs73013176 | C | 11147526 | 0.011 | -0.102 | 0.019 | 2.90E-08 | 114999 | 2.41E-04 | 27.67 | FALSE |
| rs74747585 | C | 45039971 | 0.025 | -0.069 | 0.013 | 2.60E-07 | 114999 | 2.38E-04 | 27.36 | FALSE |
| rs80254170 | G | 160514283 | 0.076 | 0.039 | 0.008 | 1.30E-07 | 114999 | 2.32E-04 | 26.70 | FALSE |
| rs740516 | G | 67082962 | 0.151 | -0.029 | 0.006 | 2.80E-07 | 114999 | 2.27E-04 | 26.09 | TRUE |
| rs2229738 | T | 68562328 | 0.066 | -0.041 | 0.008 | 3.60E-08 | 114999 | 2.24E-04 | 25.77 | FALSE |
| rs4917590 | C | 112749911 | 0.078 | -0.038 | 0.008 | 8.50E-07 | 114999 | 2.23E-04 | 25.64 | FALSE |
| rs7144265 | T | 35218625 | 0.121 | 0.031 | 0.006 | 3.90E-06 | 114999 | 2.19E-04 | 25.24 | FALSE |
| rs139974673 | C | 44027885 | 0.026 | 0.064 | 0.013 | 2.50E-07 | 114999 | 2.18E-04 | 25.10 | FALSE |
| rs4750358 | T | 13485445 | 0.307 | -0.022 | 0.004 | 6.90E-07 | 114999 | 2.14E-04 | 24.56 | FALSE |
| rs1877712 | A | 219168432 | 0.561 | -0.020 | 0.004 | 4.60E-07 | 114999 | 2.10E-04 | 24.14 | FALSE |
| rs12472790 | A | 203488594 | 0.496 | 0.020 | 0.004 | 2.30E-07 | 114999 | 2.07E-04 | 23.84 | FALSE |
| rs34101748 | A | 55025546 | 0.046 | -0.048 | 0.010 | 4.50E-07 | 114999 | 2.05E-04 | 23.63 | FALSE |
| rs11057602 | C | 124852609 | 0.611 | -0.020 | 0.004 | 3.80E-07 | 114999 | 2.05E-04 | 23.56 | FALSE |
| rs335321 | A | 62390461 | 0.733 | -0.022 | 0.005 | 2.20E-06 | 114999 | 2.03E-04 | 23.33 | FALSE |
| rs11667604 | A | 449252 | 0.399 | -0.020 | 0.004 | 1.60E-06 | 114999 | 2.02E-04 | 23.25 | TRUE |
| rs612194 | A | 37442707 | 0.252 | -0.022 | 0.005 | 1.20E-06 | 114999 | 2.01E-04 | 23.17 | TRUE |
| rs7503952 | C | 1649259 | 0.218 | 0.024 | 0.005 | 1.20E-06 | 114999 | 2.00E-04 | 23.06 | TRUE |
| rs6805251 | C | 119560606 | 0.615 | -0.020 | 0.004 | 2.30E-06 | 114999 | 1.99E-04 | 22.93 | FALSE |
| rs150688657 | A | 7505801 | 0.103 | 0.032 | 0.007 | 6.00E-07 | 114999 | 1.99E-04 | 22.88 | FALSE |
| rs11563251 | T | 234679384 | 0.111 | 0.031 | 0.006 | 1.20E-06 | 114999 | 1.99E-04 | 22.87 | FALSE |
| rs56233644 | A | 38352758 | 0.366 | -0.020 | 0.004 | 1.20E-06 | 114999 | 1.99E-04 | 22.86 | FALSE |
| rs9343614 | G | 78142473 | 0.222 | 0.023 | 0.005 | 4.30E-06 | 114999 | 1.98E-04 | 22.83 | FALSE |
| rs7610260 | T | 195682526 | 0.678 | -0.021 | 0.004 | 4.30E-06 | 114999 | 1.98E-04 | 22.76 | TRUE |
| rs35472563 | G | 219543091 | 0.021 | 0.067 | 0.014 | 1.70E-06 | 114999 | 1.98E-04 | 22.72 | FALSE |
| rs7221813 | C | 77650814 | 0.027 | 0.060 | 0.013 | 1.50E-06 | 114999 | 1.95E-04 | 22.45 | FALSE |
| rs2862954 | C | 101912064 | 0.495 | 0.019 | 0.004 | 1.60E-06 | 114999 | 1.95E-04 | 22.42 | FALSE |
| rs3808348 | T | 1028448 | 0.207 | -0.023 | 0.005 | 3.40E-07 | 114999 | 1.94E-04 | 22.32 | FALSE |
| rs11621792 | T | 24871926 | 0.454 | 0.019 | 0.004 | 1.20E-06 | 114999 | 1.91E-04 | 22.02 | FALSE |
| rs12683162 | C | 6649604 | 0.174 | 0.025 | 0.005 | 1.50E-06 | 114999 | 1.91E-04 | 21.98 | TRUE |
| rs7337784 | C | 32957972 | 0.214 | -0.023 | 0.005 | 1.80E-06 | 114999 | 1.90E-04 | 21.85 | TRUE |
| rs13098031 | T | 12239931 | 0.269 | -0.021 | 0.005 | 8.40E-07 | 114999 | 1.90E-04 | 21.80 | FALSE |
| rs12369145 | G | 125240154 | 0.090 | -0.033 | 0.007 | 1.20E-06 | 114999 | 1.89E-04 | 21.78 | FALSE |
| rs56819515 | A | 170713522 | 0.127 | 0.028 | 0.006 | 2.60E-06 | 114999 | 1.87E-04 | 21.49 | TRUE |
| rs2066714 | C | 107586753 | 0.129 | 0.028 | 0.006 | 2.10E-06 | 114999 | 1.87E-04 | 21.48 | FALSE |
| rs17018974 | A | 4344538 | 0.153 | 0.026 | 0.006 | 4.20E-06 | 114999 | 1.85E-04 | 21.25 | TRUE |
| rs6854749 | T | 87968798 | 0.198 | 0.023 | 0.005 | 2.40E-06 | 114999 | 1.85E-04 | 21.25 | TRUE |
| rs932206 | T | 136825272 | 0.641 | -0.019 | 0.004 | 3.50E-06 | 114999 | 1.84E-04 | 21.21 | FALSE |
| rs34120986 | A | 11692128 | 0.372 | -0.020 | 0.004 | 2.90E-06 | 114999 | 1.84E-04 | 21.19 | FALSE |
| rs4984741 | A | 1025464 | 0.255 | -0.021 | 0.005 | 2.60E-06 | 114999 | 1.82E-04 | 20.94 | FALSE |
| rs4782568 | G | 83980529 | 0.452 | -0.019 | 0.004 | 1.60E-06 | 114999 | 1.81E-04 | 20.83 | TRUE |
| rs1247030 | T | 161376163 | 0.111 | 0.029 | 0.006 | 2.30E-06 | 114999 | 1.80E-04 | 20.69 | FALSE |
| rs34859751 | A | 242611547 | 0.190 | 0.024 | 0.005 | 3.80E-06 | 114999 | 1.79E-04 | 20.64 | FALSE |
| rs8134638 | C | 40644170 | 0.375 | 0.019 | 0.004 | 1.20E-06 | 114999 | 1.79E-04 | 20.62 | FALSE |
| rs9564985 | A | 74002167 | 0.176 | -0.024 | 0.005 | 2.50E-06 | 114999 | 1.79E-04 | 20.55 | FALSE |
| rs34751933 | A | 180410789 | 0.093 | 0.032 | 0.007 | 3.70E-06 | 114999 | 1.78E-04 | 20.50 | FALSE |
| rs7179095 | A | 91186461 | 0.167 | 0.024 | 0.005 | 2.70E-06 | 114999 | 1.78E-04 | 20.45 | FALSE |
| rs80037662 | A | 144322988 | 0.226 | 0.022 | 0.005 | 2.00E-06 | 114999 | 1.75E-04 | 20.07 | TRUE |
| rs12196970 | T | 27311711 | 0.075 | 0.034 | 0.008 | 2.20E-06 | 114999 | 1.73E-04 | 19.93 | FALSE |
| rs11202168 | A | 88522408 | 0.297 | -0.020 | 0.004 | 3.50E-06 | 114999 | 1.73E-04 | 19.90 | FALSE |
| rs4683708 | T | 142653145 | 0.533 | -0.018 | 0.004 | 4.40E-06 | 114999 | 1.72E-04 | 19.74 | FALSE |
| **Retinol** | | | | | | | | | | |
| **SNP** | **EA** | **Position** | **EAF** | **BETA** | **SE** | **P** | **N** | **R2** | **F** | **palindromic** |
| rs117669768 | A | 100960862 | 0.038 | 0.079 | 0.015 | 9.00E-08 | 62991 | 4.54E-04 | 28.58 | FALSE |
| rs74977546 | A | 72038130 | 0.053 | -0.064 | 0.013 | 5.50E-07 | 62991 | 3.98E-04 | 25.10 | FALSE |
| rs2126371 | T | 82810664 | 0.318 | -0.029 | 0.006 | 1.30E-06 | 62991 | 3.72E-04 | 23.45 | FALSE |
| rs692790 | C | 22044929 | 0.874 | 0.040 | 0.008 | 1.40E-06 | 62991 | 3.69E-04 | 23.25 | FALSE |
| rs149577802 | T | 163575879 | 0.015 | -0.109 | 0.023 | 3.40E-06 | 62991 | 3.43E-04 | 21.59 | FALSE |
| rs3213829 | G | 61893898 | 0.546 | 0.026 | 0.006 | 3.60E-06 | 62991 | 3.40E-04 | 21.44 | FALSE |
| rs117219913 | C | 61692702 | 0.078 | 0.048 | 0.010 | 4.40E-06 | 62991 | 3.35E-04 | 21.10 | FALSE |
| rs909570 | A | 7924733 | 0.939 | -0.053 | 0.012 | 4.40E-06 | 62991 | 3.35E-04 | 21.08 | FALSE |
| **Total bilirubin** | | | | | | | | | | |
| **SNP** | **EA** | **Position** | **EAF** | **BETA** | **SE** | **P** | **N** | **R2** | **F** | **palindromic** |
| rs190591485 | C | 234490058 | 0.036 | 2.329 | 0.030 | 1.00E-200 | 342829 | 1.71E-02 | 5973.86 | FALSE |
| rs149767042 | A | 234520774 | 0.028 | 2.340 | 0.031 | 1.00E-200 | 342829 | 1.60E-02 | 5578.26 | FALSE |
| rs55781538 | C | 234637625 | 0.022 | 2.492 | 0.036 | 1.00E-200 | 342829 | 1.41E-02 | 4907.29 | FALSE |
| rs62192912 | T | 234144942 | 0.305 | -0.713 | 0.011 | 1.00E-200 | 342829 | 1.13E-02 | 3906.91 | FALSE |
| rs17862876 | T | 234657512 | 0.017 | 2.446 | 0.039 | 1.00E-200 | 342829 | 1.11E-02 | 3859.45 | FALSE |
| rs143663553 | G | 234475234 | 0.015 | 2.513 | 0.046 | 1.00E-200 | 342829 | 8.71E-03 | 3010.72 | TRUE |
| rs4149056 | C | 21331549 | 0.151 | 0.624 | 0.014 | 1.00E-200 | 342829 | 5.42E-03 | 1868.29 | FALSE |
| rs75130623 | T | 234630477 | 0.019 | -1.325 | 0.042 | 1.00E-200 | 342829 | 2.95E-03 | 1012.97 | FALSE |
| rs12814270 | G | 21097329 | 0.221 | 0.280 | 0.013 | 1.96E-110 | 342829 | 1.45E-03 | 498.90 | FALSE |
| rs17869072 | C | 234918905 | 0.149 | 0.292 | 0.015 | 7.27E-89 | 342829 | 1.16E-03 | 399.67 | FALSE |
| rs17476364 | C | 71094504 | 0.110 | 0.310 | 0.017 | 6.46E-78 | 342829 | 1.02E-03 | 349.32 | FALSE |
| rs687339 | T | 135932359 | 0.772 | -0.193 | 0.012 | 3.92E-55 | 342829 | 7.13E-04 | 244.68 | FALSE |
| rs1896995 | T | 65365385 | 0.485 | -0.137 | 0.010 | 2.41E-39 | 342829 | 5.02E-04 | 172.25 | FALSE |
| rs7310615 | G | 111865049 | 0.518 | -0.132 | 0.010 | 1.29E-36 | 342829 | 4.66E-04 | 159.77 | TRUE |
| rs450244 | C | 2940492 | 0.911 | -0.212 | 0.018 | 2.68E-31 | 342829 | 3.95E-04 | 135.44 | FALSE |
| rs2068888 | A | 94839642 | 0.449 | 0.121 | 0.010 | 4.27E-31 | 342829 | 3.92E-04 | 134.50 | FALSE |
| rs1800562 | A | 26093141 | 0.079 | 0.221 | 0.019 | 1.80E-30 | 342829 | 3.84E-04 | 131.66 | FALSE |
| rs76384978 | G | 234139631 | 0.014 | 0.500 | 0.045 | 2.93E-29 | 342829 | 3.68E-04 | 126.12 | FALSE |
| rs4410790 | C | 17284577 | 0.634 | -0.120 | 0.011 | 5.05E-29 | 342829 | 3.65E-04 | 125.02 | FALSE |
| rs1848984 | T | 94063651 | 0.188 | 0.142 | 0.013 | 9.73E-27 | 342829 | 3.34E-04 | 114.59 | TRUE |
| rs7312591 | C | 21663784 | 0.927 | -0.192 | 0.020 | 6.99E-22 | 342829 | 2.70E-04 | 92.43 | TRUE |
| rs857720 | C | 158611797 | 0.267 | -0.111 | 0.012 | 2.48E-21 | 342829 | 2.62E-04 | 89.93 | FALSE |
| rs11635675 | G | 63793238 | 0.343 | 0.102 | 0.011 | 7.83E-21 | 342829 | 2.56E-04 | 87.66 | FALSE |
| rs12414160 | G | 122872599 | 0.087 | -0.167 | 0.018 | 7.76E-20 | 342829 | 2.42E-04 | 83.12 | FALSE |
| rs34265667 | A | 41542093 | 0.035 | 0.255 | 0.028 | 2.47E-19 | 342829 | 2.36E-04 | 80.83 | FALSE |
| rs340005 | A | 60878030 | 0.621 | -0.094 | 0.011 | 8.41E-19 | 342829 | 2.29E-04 | 78.41 | FALSE |
| rs1800961 | T | 43042364 | 0.031 | 0.262 | 0.030 | 1.07E-18 | 342829 | 2.27E-04 | 77.93 | FALSE |
| rs6000553 | G | 37470224 | 0.533 | 0.091 | 0.010 | 2.48E-18 | 342829 | 2.22E-04 | 76.28 | FALSE |
| rs964184 | C | 116648917 | 0.868 | -0.131 | 0.015 | 1.39E-17 | 342829 | 2.13E-04 | 72.87 | TRUE |
| rs4475971 | A | 20734763 | 0.561 | -0.087 | 0.011 | 1.35E-16 | 342829 | 1.99E-04 | 68.39 | FALSE |
| rs9410397 | C | 91514697 | 0.057 | 0.187 | 0.023 | 1.60E-16 | 342829 | 1.98E-04 | 68.05 | FALSE |
| rs1047891 | A | 211540507 | 0.316 | 0.090 | 0.011 | 7.13E-16 | 342829 | 1.90E-04 | 65.10 | FALSE |
| rs7947951 | G | 13356030 | 0.691 | -0.090 | 0.011 | 8.94E-16 | 342829 | 1.89E-04 | 64.66 | FALSE |
| rs499974 | A | 75455021 | 0.156 | 0.114 | 0.014 | 1.71E-15 | 342829 | 1.85E-04 | 63.37 | FALSE |
| rs114165349 | C | 27021913 | 0.024 | 0.269 | 0.034 | 5.18E-15 | 342829 | 1.78E-04 | 61.20 | TRUE |
| rs1874121 | T | 220969049 | 0.340 | 0.085 | 0.011 | 8.88E-15 | 342829 | 1.75E-04 | 60.14 | FALSE |
| rs2587534 | A | 234849339 | 0.518 | 0.079 | 0.010 | 3.48E-14 | 342829 | 1.68E-04 | 57.45 | FALSE |
| rs79260437 | T | 235029651 | 0.010 | -0.396 | 0.053 | 5.16E-14 | 342829 | 1.65E-04 | 56.67 | FALSE |
| rs662138 | G | 160564476 | 0.187 | -0.099 | 0.013 | 9.89E-14 | 342829 | 1.62E-04 | 55.39 | TRUE |
| rs6750559 | A | 113841532 | 0.390 | -0.079 | 0.011 | 9.95E-14 | 342829 | 1.62E-04 | 55.38 | FALSE |
| rs55649657 | G | 21607283 | 0.220 | 0.092 | 0.013 | 2.28E-13 | 342829 | 1.57E-04 | 53.75 | TRUE |
| rs59616136 | A | 17252041 | 0.093 | 0.132 | 0.018 | 5.28E-13 | 342829 | 1.52E-04 | 52.10 | FALSE |
| rs7222046 | G | 7806529 | 0.431 | 0.075 | 0.011 | 1.41E-12 | 342829 | 1.46E-04 | 50.18 | FALSE |
| rs6750065 | T | 64889908 | 0.735 | 0.084 | 0.012 | 1.44E-12 | 342829 | 1.46E-04 | 50.13 | FALSE |
| rs2670430 | G | 69689122 | 0.878 | 0.112 | 0.016 | 1.74E-12 | 342829 | 1.45E-04 | 49.76 | TRUE |
| rs1292043 | G | 57934169 | 0.202 | -0.089 | 0.013 | 4.54E-12 | 342829 | 1.40E-04 | 47.88 | FALSE |
| rs7907226 | C | 96397386 | 0.154 | -0.097 | 0.014 | 1.35E-11 | 342829 | 1.33E-04 | 45.73 | TRUE |
| rs2968478 | G | 88858646 | 0.581 | -0.072 | 0.011 | 1.43E-11 | 342829 | 1.33E-04 | 45.63 | FALSE |
| rs1762486 | A | 107627697 | 0.656 | 0.075 | 0.011 | 1.58E-11 | 342829 | 1.33E-04 | 45.44 | FALSE |
| rs635634 | T | 136155000 | 0.184 | -0.091 | 0.013 | 1.64E-11 | 342829 | 1.32E-04 | 45.36 | FALSE |
| rs7139079 | A | 121415293 | 0.595 | 0.071 | 0.011 | 1.69E-11 | 342829 | 1.32E-04 | 45.31 | FALSE |
| rs113117433 | T | 235225025 | 0.020 | -0.253 | 0.038 | 4.11E-11 | 342829 | 1.27E-04 | 43.56 | FALSE |
| rs102275 | C | 61557803 | 0.352 | -0.071 | 0.011 | 5.93E-11 | 342829 | 1.25E-04 | 42.85 | FALSE |
| rs2296804 | G | 42931261 | 0.588 | -0.068 | 0.011 | 8.99E-11 | 342829 | 1.23E-04 | 42.04 | TRUE |
| rs5112 | G | 45430280 | 0.532 | 0.072 | 0.011 | 9.36E-11 | 342829 | 1.22E-04 | 41.95 | TRUE |
| rs7526446 | A | 63167984 | 0.637 | 0.070 | 0.011 | 1.63E-10 | 342829 | 1.19E-04 | 40.88 | FALSE |
| rs2792703 | T | 113931690 | 0.721 | -0.073 | 0.012 | 2.18E-10 | 342829 | 1.18E-04 | 40.30 | FALSE |
| rs10023050 | G | 88064431 | 0.394 | 0.067 | 0.011 | 2.57E-10 | 342829 | 1.17E-04 | 39.98 | FALSE |
| rs17316633 | A | 110825634 | 0.264 | 0.074 | 0.012 | 2.58E-10 | 342829 | 1.17E-04 | 39.97 | FALSE |
| rs13201752 | G | 28331127 | 0.319 | -0.070 | 0.011 | 2.99E-10 | 342829 | 1.16E-04 | 39.69 | FALSE |
| rs1474865 | C | 76118887 | 0.117 | -0.101 | 0.016 | 4.75E-10 | 342829 | 1.13E-04 | 38.77 | TRUE |
| rs181207 | T | 28513530 | 0.336 | -0.068 | 0.011 | 5.54E-10 | 342829 | 1.12E-04 | 38.48 | FALSE |
| rs4925546 | G | 247602968 | 0.630 | -0.067 | 0.011 | 6.01E-10 | 342829 | 1.12E-04 | 38.32 | FALSE |
| rs1688264 | G | 49209560 | 0.534 | 0.064 | 0.010 | 1.14E-09 | 342829 | 1.08E-04 | 37.08 | FALSE |
| rs4791212 | T | 65975385 | 0.204 | -0.078 | 0.013 | 1.25E-09 | 342829 | 1.08E-04 | 36.90 | FALSE |
| rs1807609 | C | 29131129 | 0.699 | 0.069 | 0.011 | 1.41E-09 | 342829 | 1.07E-04 | 36.66 | FALSE |
| rs6822348 | T | 100053894 | 0.698 | -0.067 | 0.011 | 3.22E-09 | 342829 | 1.02E-04 | 35.05 | TRUE |
| rs6129760 | G | 39746403 | 0.323 | 0.066 | 0.011 | 3.38E-09 | 342829 | 1.02E-04 | 34.95 | FALSE |
| rs2522051 | C | 131797578 | 0.454 | -0.061 | 0.010 | 4.63E-09 | 342829 | 1.00E-04 | 34.34 | FALSE |
| rs9438901 | A | 25584976 | 0.858 | 0.086 | 0.015 | 6.06E-09 | 342829 | 9.86E-05 | 33.81 | FALSE |
| rs6943025 | A | 98037557 | 0.858 | 0.087 | 0.015 | 7.30E-09 | 342829 | 9.76E-05 | 33.45 | TRUE |
| rs705753 | G | 202255929 | 0.526 | 0.060 | 0.010 | 7.95E-09 | 342829 | 9.71E-05 | 33.29 | TRUE |
| rs9482771 | C | 127446610 | 0.495 | 0.059 | 0.010 | 1.25E-08 | 342829 | 9.45E-05 | 32.40 | TRUE |
| rs76895963 | G | 4384844 | 0.021 | 0.225 | 0.040 | 1.50E-08 | 342829 | 9.35E-05 | 32.06 | FALSE |
| rs1392436 | G | 122835138 | 0.331 | 0.062 | 0.011 | 1.98E-08 | 342829 | 9.19E-05 | 31.52 | FALSE |
| rs964653 | A | 28194261 | 0.080 | -0.107 | 0.019 | 2.07E-08 | 342829 | 9.17E-05 | 31.43 | TRUE |
| rs6802898 | T | 12391207 | 0.122 | -0.088 | 0.016 | 2.49E-08 | 342829 | 9.06E-05 | 31.07 | FALSE |
| rs72681698 | C | 51207741 | 0.011 | 0.272 | 0.050 | 4.33E-08 | 342829 | 8.75E-05 | 30.00 | FALSE |
| rs11124287 | A | 32916683 | 0.663 | 0.060 | 0.011 | 4.37E-08 | 342829 | 8.74E-05 | 29.98 | FALSE |
| rs10876376 | A | 53261822 | 0.548 | 0.057 | 0.010 | 4.49E-08 | 342829 | 8.73E-05 | 29.92 | FALSE |
| rs35115456 | T | 104386842 | 0.052 | -0.128 | 0.024 | 7.04E-08 | 342829 | 8.47E-05 | 29.06 | FALSE |
| rs2255319 | T | 30539782 | 0.513 | -0.056 | 0.010 | 7.44E-08 | 342829 | 8.44E-05 | 28.95 | TRUE |
| rs11727331 | A | 145091680 | 0.052 | 0.127 | 0.024 | 8.07E-08 | 342829 | 8.40E-05 | 28.79 | FALSE |
| rs3768321 | T | 40035928 | 0.197 | -0.070 | 0.013 | 9.25E-08 | 342829 | 8.32E-05 | 28.53 | FALSE |
| rs11224309 | G | 100463238 | 0.097 | 0.093 | 0.017 | 1.04E-07 | 342829 | 8.25E-05 | 28.29 | TRUE |
| rs2535613 | G | 15868225 | 0.630 | -0.059 | 0.011 | 1.12E-07 | 342829 | 8.21E-05 | 28.15 | FALSE |
| rs6926614 | A | 126965321 | 0.808 | -0.070 | 0.013 | 1.31E-07 | 342829 | 8.12E-05 | 27.85 | FALSE |
| rs7047279 | C | 97249230 | 0.568 | 0.055 | 0.011 | 1.35E-07 | 342829 | 8.11E-05 | 27.79 | FALSE |
| rs536899661 | A | 158354839 | 0.047 | 0.134 | 0.025 | 1.47E-07 | 342829 | 8.06E-05 | 27.64 | FALSE |
| rs79485702 | G | 154679979 | 0.289 | 0.062 | 0.012 | 1.48E-07 | 342829 | 8.06E-05 | 27.62 | FALSE |
| rs73169738 | G | 170747095 | 0.078 | 0.102 | 0.019 | 1.59E-07 | 342829 | 8.01E-05 | 27.48 | FALSE |
| rs34755157 | T | 137113606 | 0.170 | -0.073 | 0.014 | 1.65E-07 | 342829 | 7.99E-05 | 27.41 | FALSE |
| rs2321536 | T | 156791268 | 0.426 | -0.058 | 0.011 | 1.68E-07 | 342829 | 7.98E-05 | 27.37 | FALSE |
| rs56389940 | A | 81259152 | 0.324 | -0.058 | 0.011 | 1.86E-07 | 342829 | 7.92E-05 | 27.17 | FALSE |
| rs5023360 | A | 124775931 | 0.109 | -0.087 | 0.017 | 2.02E-07 | 342829 | 7.88E-05 | 27.01 | TRUE |
| rs10881578 | G | 137232535 | 0.285 | -0.060 | 0.011 | 2.17E-07 | 342829 | 7.84E-05 | 26.88 | FALSE |
| rs7770144 | A | 6959748 | 0.456 | -0.054 | 0.010 | 2.22E-07 | 342829 | 7.83E-05 | 26.84 | FALSE |
| rs9826148 | T | 114464858 | 0.109 | -0.086 | 0.017 | 2.62E-07 | 342829 | 7.73E-05 | 26.51 | FALSE |
| rs1012384 | A | 59196064 | 0.827 | 0.070 | 0.014 | 2.84E-07 | 342829 | 7.69E-05 | 26.36 | FALSE |
| rs4858819 | T | 48478088 | 0.263 | 0.060 | 0.012 | 3.08E-07 | 342829 | 7.64E-05 | 26.20 | FALSE |
| rs4695913 | A | 175086260 | 0.532 | -0.053 | 0.010 | 3.18E-07 | 342829 | 7.62E-05 | 26.14 | FALSE |
| rs10733608 | T | 117148430 | 0.486 | -0.053 | 0.010 | 3.45E-07 | 342829 | 7.58E-05 | 25.98 | FALSE |
| rs11121 | T | 27359887 | 0.498 | -0.053 | 0.010 | 3.57E-07 | 342829 | 7.56E-05 | 25.92 | FALSE |
| rs2235567 | A | 6652718 | 0.360 | -0.055 | 0.011 | 3.67E-07 | 342829 | 7.54E-05 | 25.86 | FALSE |
| rs987469 | G | 89706643 | 0.461 | 0.053 | 0.010 | 3.77E-07 | 342829 | 7.53E-05 | 25.81 | TRUE |
| rs9616 | T | 154555733 | 0.296 | 0.057 | 0.011 | 4.65E-07 | 342829 | 7.41E-05 | 25.41 | TRUE |
| rs11656978 | A | 53383769 | 0.230 | -0.062 | 0.012 | 4.87E-07 | 342829 | 7.38E-05 | 25.32 | FALSE |
| rs738409 | G | 44324727 | 0.216 | 0.063 | 0.013 | 5.08E-07 | 342829 | 7.36E-05 | 25.23 | TRUE |
| rs61061000 | T | 19283268 | 0.111 | 0.083 | 0.017 | 5.85E-07 | 342829 | 7.28E-05 | 24.96 | FALSE |
| rs8041523 | T | 73982556 | 0.426 | 0.052 | 0.010 | 6.14E-07 | 342829 | 7.25E-05 | 24.87 | FALSE |
| rs10495928 | G | 46353166 | 0.336 | -0.055 | 0.011 | 6.68E-07 | 342829 | 7.21E-05 | 24.71 | FALSE |
| rs4874171 | T | 144692900 | 0.632 | -0.054 | 0.011 | 6.85E-07 | 342829 | 7.19E-05 | 24.66 | FALSE |
| rs6987080 | G | 131026459 | 0.435 | 0.052 | 0.010 | 7.17E-07 | 342829 | 7.17E-05 | 24.57 | TRUE |
| rs9971862 | G | 21740657 | 0.222 | -0.062 | 0.013 | 7.25E-07 | 342829 | 7.16E-05 | 24.55 | FALSE |
| rs1810504 | C | 17845072 | 0.846 | 0.071 | 0.014 | 7.46E-07 | 342829 | 7.14E-05 | 24.49 | TRUE |
| rs11495610 | T | 66379926 | 0.213 | -0.063 | 0.013 | 8.66E-07 | 342829 | 7.06E-05 | 24.21 | FALSE |
| rs55974805 | T | 133771130 | 0.245 | 0.059 | 0.012 | 1.08E-06 | 342829 | 6.93E-05 | 23.77 | FALSE |
| rs62020701 | C | 43259081 | 0.098 | -0.085 | 0.017 | 1.08E-06 | 342829 | 6.93E-05 | 23.77 | TRUE |
| rs73971708 | G | 170580483 | 0.171 | 0.067 | 0.014 | 1.10E-06 | 342829 | 6.93E-05 | 23.75 | FALSE |
| rs3735964 | A | 19824045 | 0.106 | -0.082 | 0.017 | 1.18E-06 | 342829 | 6.89E-05 | 23.62 | FALSE |
| rs6762415 | G | 133478557 | 0.535 | -0.051 | 0.010 | 1.16E-06 | 342829 | 6.89E-05 | 23.64 | FALSE |
| rs72729610 | G | 154190965 | 0.167 | -0.067 | 0.014 | 1.27E-06 | 342829 | 6.84E-05 | 23.46 | FALSE |
| rs16947051 | C | 61790917 | 0.337 | -0.053 | 0.011 | 1.39E-06 | 342829 | 6.80E-05 | 23.30 | TRUE |
| rs55889152 | T | 75310316 | 0.102 | 0.082 | 0.017 | 1.56E-06 | 342829 | 6.73E-05 | 23.08 | FALSE |
| rs28910284 | A | 43140578 | 0.266 | 0.056 | 0.012 | 1.67E-06 | 342829 | 6.69E-05 | 22.94 | FALSE |
| rs2087826 | A | 145639318 | 0.535 | 0.050 | 0.010 | 1.70E-06 | 342829 | 6.68E-05 | 22.91 | FALSE |
| rs62088207 | T | 73717235 | 0.157 | 0.068 | 0.014 | 1.87E-06 | 342829 | 6.63E-05 | 22.72 | FALSE |
| rs7205582 | A | 4231609 | 0.391 | 0.051 | 0.011 | 1.87E-06 | 342829 | 6.63E-05 | 22.72 | FALSE |
| rs78480755 | G | 41687828 | 0.041 | -0.125 | 0.026 | 1.96E-06 | 342829 | 6.60E-05 | 22.64 | FALSE |
| rs1519818 | A | 78541745 | 0.609 | 0.051 | 0.011 | 2.03E-06 | 342829 | 6.58E-05 | 22.57 | FALSE |
| rs4945881 | A | 111791463 | 0.185 | -0.063 | 0.013 | 2.06E-06 | 342829 | 6.57E-05 | 22.54 | FALSE |
| rs76083814 | T | 38010436 | 0.094 | 0.085 | 0.018 | 2.06E-06 | 342829 | 6.57E-05 | 22.54 | FALSE |
| rs372338580 | T | 108512843 | 0.057 | 0.107 | 0.023 | 2.25E-06 | 342829 | 6.53E-05 | 22.37 | FALSE |
| rs7213285 | A | 27206029 | 0.164 | 0.066 | 0.014 | 2.24E-06 | 342829 | 6.53E-05 | 22.38 | FALSE |
| rs13092376 | C | 196516288 | 0.419 | 0.050 | 0.011 | 2.29E-06 | 342829 | 6.51E-05 | 22.33 | FALSE |
| rs3851298 | C | 203665047 | 0.902 | -0.082 | 0.017 | 2.39E-06 | 342829 | 6.49E-05 | 22.26 | FALSE |
| rs2657878 | T | 56866962 | 0.179 | 0.064 | 0.014 | 2.42E-06 | 342829 | 6.48E-05 | 22.23 | FALSE |
| rs34473081 | A | 111112654 | 0.574 | 0.050 | 0.011 | 2.47E-06 | 342829 | 6.47E-05 | 22.19 | FALSE |
| rs61984409 | C | 64730021 | 0.362 | -0.051 | 0.011 | 2.59E-06 | 342829 | 6.45E-05 | 22.10 | FALSE |
| rs2010127 | C | 19442305 | 0.829 | -0.065 | 0.014 | 2.72E-06 | 342829 | 6.42E-05 | 22.01 | FALSE |
| rs1231204 | C | 48867273 | 0.951 | -0.111 | 0.024 | 3.13E-06 | 342829 | 6.34E-05 | 21.74 | TRUE |
| rs62396718 | C | 41633203 | 0.105 | -0.079 | 0.017 | 3.28E-06 | 342829 | 6.31E-05 | 21.65 | FALSE |
| rs11217176 | A | 119065044 | 0.270 | 0.054 | 0.012 | 3.37E-06 | 342829 | 6.30E-05 | 21.59 | FALSE |
| rs4919820 | G | 657588 | 0.844 | 0.066 | 0.014 | 3.33E-06 | 342829 | 6.30E-05 | 21.61 | FALSE |
| rs72844048 | A | 3105622 | 0.021 | 0.180 | 0.039 | 3.38E-06 | 342829 | 6.30E-05 | 21.59 | FALSE |
| rs2061298 | G | 183398861 | 0.327 | 0.051 | 0.011 | 3.74E-06 | 342829 | 6.24E-05 | 21.39 | FALSE |
| rs9923299 | T | 74263019 | 0.463 | 0.048 | 0.010 | 3.72E-06 | 342829 | 6.24E-05 | 21.40 | FALSE |
| rs10769315 | C | 48110367 | 0.789 | 0.059 | 0.013 | 3.90E-06 | 342829 | 6.22E-05 | 21.32 | FALSE |
| rs719928 | A | 101066078 | 0.070 | 0.095 | 0.021 | 4.07E-06 | 342829 | 6.19E-05 | 21.23 | FALSE |
| rs11744385 | T | 16727373 | 0.067 | -0.095 | 0.021 | 4.26E-06 | 342829 | 6.17E-05 | 21.15 | FALSE |
| rs12673943 | C | 150810763 | 0.366 | 0.050 | 0.011 | 4.44E-06 | 342829 | 6.14E-05 | 21.06 | FALSE |
| rs72835688 | T | 12811955 | 0.059 | -0.103 | 0.023 | 4.90E-06 | 342829 | 6.09E-05 | 20.88 | FALSE |
| **Vitamin C** | | | | | | | | | | |
| **SNP** | **EA** | **Position** | **EAF** | **BETA** | **SE** | **P** | **N** | **R2** | **F** | **palindromic** |
| rs4481190 | C | 155038283 | 0.351 | -0.031 | 0.006 | 9.60E-08 | 64979 | 4.38E-04 | 28.45 | FALSE |
| rs11650824 | A | 68115897 | 0.035 | 0.079 | 0.016 | 5.60E-07 | 64979 | 3.85E-04 | 25.04 | TRUE |
| rs74978963 | T | 68166615 | 0.009 | 0.151 | 0.031 | 1.20E-06 | 64979 | 3.64E-04 | 23.63 | FALSE |
| rs61868302 | T | 135260228 | 0.061 | -0.057 | 0.012 | 1.40E-06 | 64979 | 3.58E-04 | 23.26 | FALSE |
| rs1883993 | A | 25211229 | 0.095 | 0.045 | 0.009 | 1.50E-06 | 64979 | 3.55E-04 | 23.10 | FALSE |
| rs114598078 | T | 81252851 | 0.042 | 0.066 | 0.014 | 1.90E-06 | 64979 | 3.49E-04 | 22.70 | FALSE |
| rs9540734 | A | 66965411 | 0.478 | -0.026 | 0.005 | 2.30E-06 | 64979 | 3.44E-04 | 22.35 | FALSE |
| rs2018201 | G | 52385942 | 0.027 | -0.081 | 0.017 | 2.50E-06 | 64979 | 3.41E-04 | 22.16 | FALSE |
| rs17482258 | T | 26949117 | 0.099 | 0.043 | 0.009 | 3.70E-06 | 64979 | 3.29E-04 | 21.39 | FALSE |
| rs4238567 | C | 33927389 | 0.522 | 0.025 | 0.006 | 4.30E-06 | 64979 | 3.25E-04 | 21.11 | FALSE |
| rs7626478 | A | 165589571 | 0.720 | 0.028 | 0.006 | 4.50E-06 | 64979 | 3.24E-04 | 21.03 | FALSE |
| **Vitamin E** | | | | | | | | | | |
| **SNP** | **EA** | **Position** | **EAF** | **BETA** | **SE** | **P** | **N** | **R2** | **F** | **palindromic** |
| rs111306778 | A | 97282672 | 0.090 | -0.048 | 0.010 | 5.40E-07 | 64979 | 3.86E-04 | 25.11 | FALSE |
| rs71385328 | G | 51353081 | 0.011 | 0.130 | 0.026 | 7.00E-07 | 64979 | 3.79E-04 | 24.61 | FALSE |
| rs536912 | A | 109514347 | 0.736 | 0.030 | 0.006 | 9.00E-07 | 64979 | 3.71E-04 | 24.13 | FALSE |
| rs4903544 | T | 77596398 | 0.300 | -0.030 | 0.006 | 9.50E-07 | 64979 | 3.70E-04 | 24.02 | FALSE |
| rs6033 | G | 169521853 | 0.072 | -0.052 | 0.011 | 9.90E-07 | 64979 | 3.68E-04 | 23.94 | FALSE |
| rs35218694 | G | 14785162 | 0.034 | -0.074 | 0.015 | 1.30E-06 | 64979 | 3.61E-04 | 23.46 | FALSE |
| rs2723979 | G | 37364666 | 0.584 | -0.027 | 0.006 | 1.50E-06 | 64979 | 3.57E-04 | 23.18 | FALSE |
| rs79966958 | T | 37024435 | 0.013 | -0.117 | 0.025 | 2.00E-06 | 64979 | 3.48E-04 | 22.61 | FALSE |
| rs979218 | C | 9684248 | 0.098 | -0.043 | 0.009 | 3.10E-06 | 64979 | 3.35E-04 | 21.77 | FALSE |
| rs12421920 | G | 13249759 | 0.094 | -0.043 | 0.009 | 3.70E-06 | 64979 | 3.30E-04 | 21.43 | FALSE |
| rs12899673 | A | 92561644 | 0.333 | 0.027 | 0.006 | 3.80E-06 | 64979 | 3.29E-04 | 21.37 | FALSE |
| **Zinc** | | | | | | | | | | |
| **SNP** | **EA** | **Position** | **EAF** | **BETA** | **SE** | **P** | **N** | **R2** | **F** | **palindromic** |
| rs10484100 | G | 86817096 | NA | -0.209 | 0.045 | 3.30E-06 | 2603 | 8.22E-03 | 21.55 | FALSE |
| rs10931753 | C | 154568757 | NA | -0.129 | 0.028 | 4.94E-06 | 2603 | 8.09E-03 | 21.21 | TRUE |
| rs11232535 | C | 80928809 | NA | 0.325 | 0.065 | 6.73E-07 | 2603 | 9.51E-03 | 24.98 | FALSE |
| rs11763353 | G | 15630871 | NA | -0.192 | 0.039 | 6.90E-07 | 2603 | 9.23E-03 | 24.22 | FALSE |
| rs1532423 | G | 86268313 | NA | -0.178 | 0.026 | 6.40E-12 | 2603 | 1.77E-02 | 46.83 | FALSE |
| rs2120019 | C | 75334184 | NA | -0.287 | 0.033 | 1.55E-18 | 2603 | 2.82E-02 | 75.58 | FALSE |
| rs4333127 | A | 5930033 | NA | 0.218 | 0.047 | 3.00E-06 | 2603 | 8.20E-03 | 21.50 | FALSE |
| rs7148590 | A | 65473196 | NA | -0.140 | 0.026 | 1.37E-07 | 2603 | 1.10E-02 | 28.97 | FALSE |
| **Annotation:** OSIB, oxidative stress injury biomarker; SNP, single-nucleotide polymorphism; EAF, effect allele frequency; EA, effect allele; BETA, beta. exposure; SE, standard error; P, the Significance level of oxidative stress; R² was calculated as follows: 2*BETA^2*EAF*(1-EAF). If the data of EAF is missing, R² was calculated as follows: BETA^2/(BETA^+SE^2*N). The F-statistic for each SNP was calculated as follows: F =((N-2)*(R2/(1-R2)), Palindromic: SNPs with intermediate allele frequencies (>0.3) were removed | | | | | | | | | | |

| **Table S2. Characteristics of SNPs associated with lung cancer.** | | | | | | | | | | |
| --- | --- | --- | --- | --- | --- | --- | --- | --- | --- | --- |
| **Small cell lung cancer** | | | | | | | | | | |
| **SNP** | **EA** | **Position** | **EAF** | **BETA** | **SE** | **P** | **N** | **R2** | **F** | **palindromic** |
| rs117969939 | T | 80389492 | 0.03 | 1.58 | 0.34 | 2.56E-06 | 218792 | 1.01E-04 | 22.12 | FALSE |
| rs17111652 | T | 55590465 | 0.07 | 1.10 | 0.23 | 1.60E-06 | 218792 | 1.05E-04 | 23.01 | FALSE |
| rs28433440 | G | 125723713 | 0.34 | 0.53 | 0.11 | 3.96E-06 | 218792 | 9.73E-05 | 21.30 | FALSE |
| rs2920066 | G | 108107927 | 0.71 | -0.55 | 0.12 | 4.44E-06 | 218792 | 9.62E-05 | 21.05 | TRUE |
| rs62581702 | T | 112468745 | 0.14 | 0.75 | 0.16 | 2.88E-06 | 218792 | 1.00E-04 | 21.89 | TRUE |
| rs75203807 | G | 42086813 | 0.03 | 1.61 | 0.35 | 3.79E-06 | 218792 | 9.76E-05 | 21.37 | FALSE |
| rs755204 | A | 61994165 | 0.08 | 1.07 | 0.21 | 3.22E-07 | 218792 | 1.19E-04 | 26.11 | FALSE |
| rs77757854 | A | 20904029 | 0.03 | 1.63 | 0.34 | 2.05E-06 | 218792 | 1.03E-04 | 22.55 | FALSE |
| rs847648 | T | 102553850 | 0.33 | 0.54 | 0.11 | 2.14E-06 | 218792 | 1.03E-04 | 22.45 | FALSE |
| rs9983414 | C | 39184876 | 0.77 | -0.62 | 0.13 | 1.81E-06 | 218792 | 1.04E-04 | 22.77 | TRUE |
| **Squamous** | | | | | | | | | | |
| **SNP** | **EA** | **Position** | **EAF** | **BETA** | **SE** | **P** | **N** | **R2** | **F** | **palindromic** |
| rs112888804 | A | 6110057 | 0.09 | 0.68 | 0.14 | 1.26E-06 | 218792 | 1.07E-04 | 23.49 | FALSE |
| rs116743496 | A | 10528439 | 0.03 | 1.07 | 0.23 | 4.11E-06 | 218792 | 9.70E-05 | 21.22 | TRUE |
| rs147130851 | A | 144880015 | 0.03 | 1.27 | 0.26 | 1.47E-06 | 218792 | 1.06E-04 | 23.19 | FALSE |
| rs150087481 | A | 102906892 | 0.01 | 1.78 | 0.39 | 4.34E-06 | 218792 | 9.64E-05 | 21.10 | FALSE |
| rs183413045 | G | 14228875 | 0.00 | 4.87 | 0.97 | 4.73E-07 | 218792 | 1.16E-04 | 25.37 | TRUE |
| rs28590060 | A | 79052312 | 0.33 | 0.40 | 0.08 | 4.60E-07 | 218792 | 1.16E-04 | 25.40 | FALSE |
| rs2873673 | A | 146723688 | 0.99 | -2.18 | 0.47 | 2.95E-06 | 218792 | 9.99E-05 | 21.85 | FALSE |
| rs3125559 | T | 133177467 | 0.09 | -0.64 | 0.14 | 2.66E-06 | 218792 | 1.01E-04 | 22.05 | FALSE |
| rs35487577 | G | 58019576 | 0.01 | 1.90 | 0.40 | 1.68E-06 | 218792 | 1.05E-04 | 22.93 | FALSE |
| rs7174390 | C | 58025960 | 0.55 | -0.38 | 0.08 | 5.54E-07 | 218792 | 1.15E-04 | 25.08 | FALSE |
| rs72909164 | G | 157717300 | 0.02 | 1.65 | 0.35 | 2.84E-06 | 218792 | 1.00E-04 | 21.93 | FALSE |
| rs73013059 | C | 129477 | 0.34 | 0.38 | 0.08 | 1.34E-06 | 218792 | 1.07E-04 | 23.39 | FALSE |
| rs75550918 | A | 66116154 | 0.00 | 4.35 | 0.92 | 1.94E-06 | 218792 | 1.04E-04 | 22.65 | FALSE |
| rs76899695 | A | 127207315 | 0.04 | 1.01 | 0.21 | 1.57E-06 | 218792 | 1.05E-04 | 23.07 | FALSE |
| rs79081142 | A | 180573814 | 0.07 | 0.77 | 0.16 | 7.07E-07 | 218792 | 1.12E-04 | 24.58 | FALSE |
| rs79521915 | A | 77167857 | 0.01 | 2.33 | 0.49 | 1.69E-06 | 218792 | 1.05E-04 | 22.92 | FALSE |
| **Adenocarcinoma** | | | | | | | | | | |
| **SNP** | **EA** | **Position** | **EAF** | **BETA** | **SE** | **P** | **N** | **R2** | **F** | **palindromic** |
| rs11009125 | G | 33174156 | 0.00 | 3.03 | 0.64 | 2.55E-06 | 218792 | 1.01E-04 | 22.13 | FALSE |
| rs111527400 | T | 105959470 | 0.04 | 0.76 | 0.16 | 3.32E-06 | 218792 | 9.89E-05 | 21.63 | FALSE |
| rs113628447 | T | 19000186 | 0.17 | 0.38 | 0.08 | 3.66E-06 | 218792 | 9.79E-05 | 21.42 | FALSE |
| rs117297323 | T | 24232572 | 0.02 | 1.20 | 0.26 | 4.05E-06 | 218792 | 9.71E-05 | 21.24 | FALSE |
| rs146930539 | G | 36231350 | 0.01 | 1.71 | 0.37 | 4.92E-06 | 218792 | 9.54E-05 | 20.86 | FALSE |
| rs148193243 | T | 44648596 | 0.03 | 0.84 | 0.18 | 4.39E-06 | 218792 | 9.63E-05 | 21.08 | FALSE |
| rs2247877 | A | 17853599 | 0.03 | 0.85 | 0.19 | 4.69E-06 | 218792 | 9.58E-05 | 20.96 | FALSE |
| rs73406452 | T | 48661984 | 0.05 | 0.74 | 0.15 | 1.37E-06 | 218792 | 1.07E-04 | 23.30 | FALSE |
| rs7414 | A | 18075053 | 0.01 | 1.26 | 0.27 | 4.02E-06 | 218792 | 9.72E-05 | 21.26 | FALSE |
| rs951266 | A | 78878541 | 0.33 | 0.30 | 0.06 | 2.57E-06 | 218792 | 1.01E-04 | 22.13 | FALSE |
| rs9905262 | G | 161436 | 0.77 | -0.35 | 0.07 | 2.20E-06 | 218792 | 1.02E-04 | 22.42 | FALSE |
| **Annotation:** SNP, single-nucleotide polymorphism; EAF, effect allele frequency; EA, effect allele; BETA, beta. exposure; SE, standard error; P, the Significance level of lung cancer; R² was calculated as follows: 2*BETA^2*EAF*(1-EAF). The F-statistic for each SNP was calculated as follows: F =((N-2)*(R2/(1-R2)), Palindromic: SNPs with intermediate allele frequencies (>0.3) were removed | | | | | | | | | | |

| **Table S3. Associations between genetically predicted adenocarcinoma and 16 OSIBs.** | | | | | | |
| --- | --- | --- | --- | --- | --- | --- |
| Exposure | Outcome | Method | Nsnp | OR (95%CI) | P | FDR |
| Adenocarcinoma | Albumin | IVW | 10 | 1.003(0.996-1.011) | 0.380 | 0.934 |
|  | Albumin | MR Egger | 10 | 1.001(0.987-1.015) | 0.909 | 0.944 |
|  | Albumin | Weighted median | 10 | 1.007(0.998-1.017) | 0.129 | 0.971 |
|  | Albumin | Weighted mode | 10 | 1.009(0.996-1.021) | 0.205 | 0.899 |
|  | Albumin | Simple mode | 10 | 1.007(0.992-1.023) | 0.389 | 0.797 |
|  | Catalase | IVW | 10 | 0.983(0.941-1.026) | 0.438 | 0.934 |
|  | Catalase | MR Egger | 10 | 0.974(0.899-1.055) | 0.532 | 0.944 |
|  | Catalase | Weighted median | 10 | 0.975(0.924-1.03) | 0.365 | 0.971 |
|  | Catalase | Weighted mode | 10 | 0.972(0.908-1.039) | 0.425 | 0.899 |
|  | Catalase | Simple mode | 10 | 0.964(0.894-1.038) | 0.357 | 0.797 |
|  | Gamma-tocopherol | IVW | 3 | 1.008(0.996-1.019) | 0.212 | 0.848 |
|  | Gamma-tocopherol | MR Egger | 3 | 1.001(0.978-1.024) | 0.944 | 0.944 |
|  | Gamma-tocopherol | Weighted median | 3 | 1.008(0.994-1.022) | 0.255 | 0.971 |
|  | Gamma-tocopherol | Weighted mode | 3 | 1.001(0.985-1.018) | 0.899 | 0.899 |
|  | Gamma-tocopherol | Simple mode | 3 | 1.014(0.995-1.033) | 0.288 | 0.797 |
|  | Glutathione peroxidase 7 | IVW | 10 | 0.991(0.947-1.037) | 0.692 | 0.944 |
|  | Glutathione peroxidase 7 | MR Egger | 10 | 0.989(0.905-1.082) | 0.822 | 0.944 |
|  | Glutathione peroxidase 7 | Weighted median | 10 | 1.001(0.943-1.063) | 0.971 | 0.971 |
|  | Glutathione peroxidase 7 | Weighted mode | 10 | 1.038(0.957-1.127) | 0.389 | 0.899 |
|  | Glutathione peroxidase 7 | Simple mode | 10 | 0.97(0.875-1.074) | 0.569 | 0.797 |
|  | Glutathione S-transferase A1 | IVW | 10 | 1.039(0.987-1.093) | 0.145 | 0.773 |
|  | Glutathione S-transferase A1 | MR Egger | 10 | 1.071(0.972-1.18) | 0.204 | 0.944 |
|  | Glutathione S-transferase A1 | Weighted median | 10 | 1.03(0.971-1.092) | 0.329 | 0.971 |
|  | Glutathione S-transferase A1 | Weighted mode | 10 | 1.028(0.965-1.096) | 0.407 | 0.899 |
|  | Glutathione S-transferase A1 | Simple mode | 10 | 1.034(0.956-1.119) | 0.423 | 0.797 |
|  | Hypoxanthine | IVW | 3 | 1.002(0.992-1.012) | 0.713 | 0.944 |
|  | Hypoxanthine | MR Egger | 3 | 1.008(0.985-1.032) | 0.607 | 0.944 |
|  | Hypoxanthine | Weighted median | 3 | 0.999(0.991-1.008) | 0.850 | 0.971 |
|  | Hypoxanthine | Weighted mode | 3 | 1.009(0.999-1.019) | 0.232 | 0.899 |
|  | Hypoxanthine | Simple mode | 3 | 0.995(0.98-1.01) | 0.586 | 0.797 |
|  | Kynurenine | IVW | 3 | 1.003(0.997-1.009) | 0.299 | 0.934 |
|  | Kynurenine | MR Egger | 3 | 0.997(0.988-1.006) | 0.595 | 0.944 |
|  | Kynurenine | Weighted median | 3 | 1.002(0.996-1.007) | 0.548 | 0.971 |
|  | Kynurenine | Weighted mode | 3 | 1.002(0.996-1.007) | 0.637 | 0.899 |
|  | Kynurenine | Simple mode | 3 | 1.002(0.995-1.008) | 0.645 | 0.797 |
|  | Kynurenine--oxoglutarate transaminase 3 | IVW | 10 | 0.993(0.951-1.037) | 0.767 | 0.944 |
|  | Kynurenine--oxoglutarate transaminase 3 | MR Egger | 10 | 1.009(0.932-1.094) | 0.826 | 0.944 |
|  | Kynurenine--oxoglutarate transaminase 3 | Weighted median | 10 | 1.014(0.957-1.075) | 0.633 | 0.971 |
|  | Kynurenine--oxoglutarate transaminase 3 | Weighted mode | 10 | 1.013(0.947-1.083) | 0.723 | 0.899 |
|  | Kynurenine--oxoglutarate transaminase 3 | Simple mode | 10 | 1.01(0.925-1.102) | 0.836 | 0.836 |
|  | Lactate | IVW | 10 | 0.997(0.99-1.005) | 0.467 | 0.934 |
|  | Lactate | MR Egger | 10 | 0.998(0.985-1.012) | 0.791 | 0.944 |
|  | Lactate | Weighted median | 10 | 1(0.99-1.009) | 0.937 | 0.971 |
|  | Lactate | Weighted mode | 10 | 0.999(0.988-1.01) | 0.821 | 0.899 |
|  | Lactate | Simple mode | 10 | 0.996(0.982-1.011) | 0.642 | 0.797 |
|  | Monounsaturated fatty acids | IVW | 10 | 1.001(0.993-1.01) | 0.757 | 0.944 |
|  | Monounsaturated fatty acids | MR Egger | 10 | 0.99(0.975-1.004) | 0.209 | 0.944 |
|  | Monounsaturated fatty acids | Weighted median | 10 | 0.999(0.989-1.009) | 0.832 | 0.971 |
|  | Monounsaturated fatty acids | Weighted mode | 10 | 0.999(0.988-1.01) | 0.886 | 0.899 |
|  | Monounsaturated fatty acids | Simple mode | 10 | 0.997(0.985-1.01) | 0.690 | 0.797 |
|  | Polyunsaturated fatty acids | IVW | 10 | 1.007(0.999-1.014) | 0.077 | 0.752 |
|  | Polyunsaturated fatty acids | MR Egger | 10 | 0.999(0.986-1.012) | 0.877 | 0.944 |
|  | Polyunsaturated fatty acids | Weighted median | 10 | 1.006(0.996-1.016) | 0.228 | 0.971 |
|  | Polyunsaturated fatty acids | Weighted mode | 10 | 1.005(0.992-1.018) | 0.456 | 0.899 |
|  | Polyunsaturated fatty acids | Simple mode | 10 | 0.996(0.979-1.013) | 0.683 | 0.797 |
|  | Retinol | IVW | 10 | 1(0.989-1.012) | 0.957 | 0.973 |
|  | Retinol | MR Egger | 10 | 1.003(0.979-1.026) | 0.836 | 0.944 |
|  | Retinol | Weighted median | 10 | 0.998(0.985-1.012) | 0.817 | 0.971 |
|  | Retinol | Weighted mode | 10 | 0.995(0.981-1.01) | 0.558 | 0.899 |
|  | Retinol | Simple mode | 10 | 0.99(0.969-1.012) | 0.388 | 0.797 |
|  | Total bilirubin | IVW | 9 | 1.001(0.982-1.02) | 0.921 | 0.973 |
|  | Total bilirubin | MR Egger | 9 | 1.014(0.979-1.051) | 0.462 | 0.944 |
|  | Total bilirubin | Weighted median | 9 | 1.006(0.981-1.032) | 0.624 | 0.971 |
|  | Total bilirubin | Weighted mode | 9 | 1.007(0.979-1.036) | 0.643 | 0.899 |
|  | Total bilirubin | Simple mode | 9 | 1.007(0.974-1.041) | 0.697 | 0.797 |
|  | Vitamin C | IVW | 10 | 1.004(0.99-1.018) | 0.622 | 0.944 |
|  | Vitamin C | MR Egger | 10 | 0.982(0.961-1.002) | 0.122 | 0.944 |
|  | Vitamin C | Weighted median | 10 | 1.006(0.991-1.022) | 0.432 | 0.971 |
|  | Vitamin C | Weighted mode | 10 | 1.013(0.983-1.044) | 0.429 | 0.899 |
|  | Vitamin C | Simple mode | 10 | 1.018(0.987-1.05) | 0.283 | 0.797 |
|  | Vitamin E | IVW | 10 | 1(0.991-1.01) | 0.973 | 0.973 |
|  | Vitamin E | MR Egger | 10 | 0.991(0.974-1.009) | 0.370 | 0.944 |
|  | Vitamin E | Weighted median | 10 | 0.999(0.986-1.012) | 0.869 | 0.971 |
|  | Vitamin E | Weighted mode | 10 | 0.995(0.98-1.011) | 0.553 | 0.899 |
|  | Vitamin E | Simple mode | 10 | 1.003(0.983-1.023) | 0.795 | 0.836 |
|  | Zinc | IVW | 3 | 0.927(0.849-1.013) | 0.094 | 0.752 |
|  | Zinc | MR Egger | 3 | 0.914(0.721-1.161) | 0.596 | 0.944 |
|  | Zinc | Weighted median | 3 | 0.925(0.845-1.013) | 0.091 | 0.971 |
|  | Zinc | Weighted mode | 3 | 0.983(0.878-1.101) | 0.800 | 0.899 |
|  | Zinc | Simple mode | 3 | 0.893(0.775-1.03) | 0.261 | 0.797 |

OSIB, oxidative stress injury biomarker; MR,mendelian randomization; SNP, single nucleotide polymorphism; IVW inverse-variance weighted; OR, odds ratio; CI, confidence interval.

| **Table S4. Associations between genetically predicted squamous and 16 OSIBs.** | | | | | | |
| --- | --- | --- | --- | --- | --- | --- |
| Exposure | Outcome | Method | Nsnp | OR (95%CI) | P | FDR |
| Squamous | Albumin | IVW | 14 | 1.001(0.996-1.005) | 0.68 | 0.975 |
|  | Albumin | MR Egger | 14 | 1.002(0.995-1.008) | 0.612 | 0.781 |
|  | Albumin | Weighted median | 14 | 1.002(0.996-1.008) | 0.573 | 0.988 |
|  | Albumin | Weighted mode | 14 | 1.001(0.994-1.008) | 0.72 | 0.949 |
|  | Albumin | Simple mode | 14 | 0.997(0.987-1.007) | 0.535 | 0.919 |
|  | Catalase | IVW | 14 | 1.012(0.984-1.04) | 0.411 | 0.975 |
|  | Catalase | MR Egger | 14 | 1.01(0.97-1.052) | 0.647 | 0.781 |
|  | Catalase | Weighted median | 14 | 1.015(0.975-1.057) | 0.464 | 0.988 |
|  | Catalase | Weighted mode | 14 | 1.016(0.976-1.058) | 0.461 | 0.949 |
|  | Catalase | Simple mode | 14 | 1.018(0.96-1.08) | 0.564 | 0.919 |
|  | Gamma-tocopherol | IVW | 3 | 1.002(0.979-1.025) | 0.876 | 0.975 |
|  | Gamma-tocopherol | MR Egger | 3 | 1.036(0.984-1.09) | 0.404 | 0.781 |
|  | Gamma-tocopherol | Weighted median | 3 | 1.002(0.98-1.025) | 0.873 | 0.988 |
|  | Gamma-tocopherol | Weighted mode | 3 | 1.003(0.978-1.029) | 0.843 | 0.949 |
|  | Gamma-tocopherol | Simple mode | 3 | 1.011(0.983-1.04) | 0.532 | 0.919 |
|  | Glutathione peroxidase 7 | IVW | 14 | 1.004(0.977-1.033) | 0.753 | 0.975 |
|  | Glutathione peroxidase 7 | MR Egger | 14 | 1.009(0.969-1.051) | 0.666 | 0.781 |
|  | Glutathione peroxidase 7 | Weighted median | 14 | 1(0.963-1.039) | 0.988 | 0.988 |
|  | Glutathione peroxidase 7 | Weighted mode | 14 | 1.002(0.958-1.048) | 0.925 | 0.949 |
|  | Glutathione peroxidase 7 | Simple mode | 14 | 1.004(0.944-1.068) | 0.902 | 0.963 |
|  | Glutathione S-transferase A1 | IVW | 14 | 1.004(0.976-1.032) | 0.793 | 0.975 |
|  | Glutathione S-transferase A1 | MR Egger | 14 | 1.009(0.969-1.051) | 0.673 | 0.781 |
|  | Glutathione S-transferase A1 | Weighted median | 14 | 1.016(0.978-1.056) | 0.414 | 0.988 |
|  | Glutathione S-transferase A1 | Weighted mode | 14 | 1.016(0.97-1.063) | 0.517 | 0.949 |
|  | Glutathione S-transferase A1 | Simple mode | 14 | 1.002(0.939-1.068) | 0.963 | 0.963 |
|  | Hypoxanthine | IVW | 3 | 1.001(0.992-1.01) | 0.826 | 0.975 |
|  | Hypoxanthine | MR Egger | 3 | 0.994(0.971-1.017) | 0.687 | 0.781 |
|  | Hypoxanthine | Weighted median | 3 | 0.997(0.987-1.008) | 0.584 | 0.988 |
|  | Hypoxanthine | Weighted mode | 3 | 0.996(0.981-1.01) | 0.618 | 0.949 |
|  | Hypoxanthine | Simple mode | 3 | 0.996(0.982-1.01) | 0.608 | 0.919 |
|  | Kynurenine | IVW | 3 | 1.001(0.994-1.008) | 0.781 | 0.975 |
|  | Kynurenine | MR Egger | 3 | 1.011(0.997-1.025) | 0.376 | 0.781 |
|  | Kynurenine | Weighted median | 3 | 1.001(0.994-1.008) | 0.814 | 0.988 |
|  | Kynurenine | Weighted mode | 3 | 0.999(0.987-1.01) | 0.822 | 0.949 |
|  | Kynurenine | Simple mode | 3 | 1.004(0.993-1.015) | 0.565 | 0.919 |
|  | Kynurenine--oxoglutarate transaminase 3 | IVW | 14 | 0.993(0.966-1.021) | 0.622 | 0.975 |
|  | Kynurenine--oxoglutarate transaminase 3 | MR Egger | 14 | 0.986(0.946-1.027) | 0.501 | 0.781 |
|  | Kynurenine--oxoglutarate transaminase 3 | Weighted median | 14 | 0.979(0.938-1.021) | 0.317 | 0.988 |
|  | Kynurenine--oxoglutarate transaminase 3 | Weighted mode | 14 | 0.968(0.927-1.01) | 0.157 | 0.949 |
|  | Kynurenine--oxoglutarate transaminase 3 | Simple mode | 14 | 0.957(0.893-1.025) | 0.234 | 0.919 |
|  | Lactate | IVW | 14 | 0.996(0.991-1.001) | 0.1 | 0.975 |
|  | Lactate | MR Egger | 14 | 0.991(0.985-0.998) | 0.021 | 0.336 |
|  | Lactate | Weighted median | 14 | 0.993(0.986-1) | 0.043 | 0.688 |
|  | Lactate | Weighted mode | 14 | 0.991(0.984-0.999) | 0.043 | 0.688 |
|  | Lactate | Simple mode | 14 | 1.003(0.99-1.016) | 0.692 | 0.923 |
|  | Monounsaturated fatty acids | IVW | 14 | 1.002(0.997-1.007) | 0.405 | 0.975 |
|  | Monounsaturated fatty acids | MR Egger | 14 | 0.999(0.992-1.006) | 0.791 | 0.791 |
|  | Monounsaturated fatty acids | Weighted median | 14 | 0.999(0.993-1.006) | 0.843 | 0.988 |
|  | Monounsaturated fatty acids | Weighted mode | 14 | 0.999(0.992-1.005) | 0.643 | 0.949 |
|  | Monounsaturated fatty acids | Simple mode | 14 | 0.998(0.988-1.007) | 0.632 | 0.919 |
|  | Polyunsaturated fatty acids | IVW | 14 | 1.003(0.999-1.008) | 0.135 | 0.975 |
|  | Polyunsaturated fatty acids | MR Egger | 14 | 1.001(0.995-1.007) | 0.732 | 0.781 |
|  | Polyunsaturated fatty acids | Weighted median | 14 | 1.002(0.996-1.008) | 0.437 | 0.988 |
|  | Polyunsaturated fatty acids | Weighted mode | 14 | 1.001(0.994-1.007) | 0.824 | 0.949 |
|  | Polyunsaturated fatty acids | Simple mode | 14 | 1.003(0.994-1.012) | 0.517 | 0.919 |
|  | Retinol | IVW | 14 | 0.997(0.991-1.003) | 0.402 | 0.975 |
|  | Retinol | MR Egger | 14 | 0.996(0.987-1.004) | 0.329 | 0.781 |
|  | Retinol | Weighted median | 14 | 0.996(0.987-1.005) | 0.359 | 0.988 |
|  | Retinol | Weighted mode | 14 | 0.995(0.985-1.005) | 0.337 | 0.949 |
|  | Retinol | Simple mode | 14 | 0.998(0.984-1.013) | 0.827 | 0.963 |
|  | Total bilirubin | IVW | 14 | 1.002(0.99-1.014) | 0.762 | 0.975 |
|  | Total bilirubin | MR Egger | 14 | 1.006(0.988-1.024) | 0.54 | 0.781 |
|  | Total bilirubin | Weighted median | 14 | 1(0.984-1.018) | 0.955 | 0.988 |
|  | Total bilirubin | Weighted mode | 14 | 1.001(0.984-1.017) | 0.949 | 0.949 |
|  | Total bilirubin | Simple mode | 14 | 1.011(0.984-1.039) | 0.448 | 0.919 |
|  | Vitamin C | IVW | 14 | 1(0.994-1.006) | 0.919 | 0.975 |
|  | Vitamin C | MR Egger | 14 | 1.003(0.994-1.012) | 0.514 | 0.781 |
|  | Vitamin C | Weighted median | 14 | 0.999(0.99-1.007) | 0.774 | 0.988 |
|  | Vitamin C | Weighted mode | 14 | 0.998(0.99-1.007) | 0.726 | 0.949 |
|  | Vitamin C | Simple mode | 14 | 0.999(0.987-1.012) | 0.916 | 0.963 |
|  | Vitamin E | IVW | 14 | 0.997(0.991-1.003) | 0.378 | 0.975 |
|  | Vitamin E | MR Egger | 14 | 1.003(0.994-1.011) | 0.532 | 0.781 |
|  | Vitamin E | Weighted median | 14 | 0.997(0.988-1.006) | 0.522 | 0.988 |
|  | Vitamin E | Weighted mode | 14 | 0.998(0.989-1.007) | 0.603 | 0.949 |
|  | Vitamin E | Simple mode | 14 | 0.997(0.984-1.009) | 0.607 | 0.919 |
|  | Zinc | IVW | 3 | 1.001(0.943-1.062) | 0.975 | 0.975 |
|  | Zinc | MR Egger | 3 | 0.953(0.873-1.041) | 0.478 | 0.781 |
|  | Zinc | Weighted median | 3 | 1.003(0.942-1.068) | 0.92 | 0.988 |
|  | Zinc | Weighted mode | 3 | 0.97(0.904-1.042) | 0.495 | 0.949 |
|  | Zinc | Simple mode | 3 | 1.061(0.95-1.186) | 0.404 | 0.919 |

OSIB, oxidative stress injury biomarker; MR,mendelian randomization; SNP, single nucleotide polymorphism; IVW inverse-variance weighted; OR, odds ratio; CI, confidence interval.

| **Table S5. Associations between genetically predicted small cell lung cancer and 16 OSIBs.** | | | | | | |
| --- | --- | --- | --- | --- | --- | --- |
| Exposure | Outcome | Method | Nsnp | OR (95%CI) | P | FDR |
| Small cell lung cancer | Albumin | IVW | 7 | 0.998(0.992-1.003） | 0.382 | 0.983 |
|  | Albumin | MR Egger | 7 | 0.998(0.985-1.011） | 0.737 | 0.954 |
|  | Albumin | Weighted median | 7 | 0.997(0.99-1.004） | 0.441 | 0.934 |
|  | Albumin | Weighted mode | 7 | 0.997(0.989-1.006） | 0.534 | 0.949 |
|  | Albumin | Simple mode | 7 | 0.997(0.987-1.008） | 0.647 | 0.966 |
|  | Catalase | IVW | 7 | 1.004(0.971-1.037） | 0.825 | 0.983 |
|  | Catalase | MR Egger | 7 | 1.035(0.963-1.112） | 0.398 | 0.91 |
|  | Catalase | Weighted median | 7 | 0.999(0.955-1.044） | 0.959 | 0.959 |
|  | Catalase | Weighted mode | 7 | 0.998(0.938-1.061） | 0.953 | 0.986 |
|  | Catalase | Simple mode | 7 | 0.983(0.916-1.056） | 0.661 | 0.966 |
|  | Gamma-tocopherol | IVW | 4 | 1.004(0.996-1.012） | 0.37 | 0.983 |
|  | Gamma-tocopherol | MR Egger | 4 | 1(0.983-1.018） | 0.961 | 0.979 |
|  | Gamma-tocopherol | Weighted median | 4 | 1.002(0.992-1.011） | 0.745 | 0.934 |
|  | Gamma-tocopherol | Weighted mode | 4 | 1.002(0.991-1.013） | 0.773 | 0.986 |
|  | Gamma-tocopherol | Simple mode | 4 | 1.002(0.99-1.014） | 0.772 | 0.966 |
|  | Glutathione peroxidase 7 | IVW | 7 | 1.028(0.995-1.062） | 0.101 | 0.983 |
|  | Glutathione peroxidase 7 | MR Egger | 7 | 1.056(0.983-1.134） | 0.194 | 0.91 |
|  | Glutathione peroxidase 7 | Weighted median | 7 | 1.026(0.983-1.072） | 0.242 | 0.934 |
|  | Glutathione peroxidase 7 | Weighted mode | 7 | 1.02(0.965-1.079） | 0.502 | 0.949 |
|  | Glutathione peroxidase 7 | Simple mode | 7 | 1.018(0.956-1.084） | 0.596 | 0.966 |
|  | Glutathione S-transferase A1 | IVW | 7 | 1.021(0.986-1.057） | 0.238 | 0.983 |
|  | Glutathione S-transferase A1 | MR Egger | 7 | 1.009(0.93-1.095） | 0.835 | 0.954 |
|  | Glutathione S-transferase A1 | Weighted median | 7 | 1.005(0.963-1.048） | 0.817 | 0.934 |
|  | Glutathione S-transferase A1 | Weighted mode | 7 | 1.001(0.955-1.05） | 0.969 | 0.986 |
|  | Glutathione S-transferase A1 | Simple mode | 7 | 1.003(0.952-1.057） | 0.912 | 0.966 |
|  | Hypoxanthine | IVW | 4 | 1(0.99-1.009） | 0.934 | 0.983 |
|  | Hypoxanthine | MR Egger | 4 | 1.015(1.006-1.025） | 0.089 | 0.91 |
|  | Hypoxanthine | Weighted median | 4 | 1.002(0.996-1.009） | 0.478 | 0.934 |
|  | Hypoxanthine | Weighted mode | 4 | 1.003(0.997-1.009） | 0.376 | 0.949 |
|  | Hypoxanthine | Simple mode | 4 | 1.002(0.995-1.009） | 0.584 | 0.966 |
|  | Kynurenine | IVW | 4 | 1.003(0.998-1.008） | 0.201 | 0.983 |
|  | Kynurenine | MR Egger | 4 | 1.007(0.996-1.018） | 0.357 | 0.91 |
|  | Kynurenine | Weighted median | 4 | 1(0.996-1.005） | 0.927 | 0.959 |
|  | Kynurenine | Weighted mode | 4 | 1(0.995-1.005） | 0.986 | 0.986 |
|  | Kynurenine | Simple mode | 4 | 1(0.994-1.006） | 0.966 | 0.966 |
|  | Kynurenine--oxoglutarate transaminase 3 | IVW | 7 | 0.999(0.953-1.048） | 0.971 | 0.983 |
|  | Kynurenine--oxoglutarate transaminase 3 | MR Egger | 7 | 0.97(0.87-1.082） | 0.61 | 0.954 |
|  | Kynurenine--oxoglutarate transaminase 3 | Weighted median | 7 | 1.012(0.961-1.065） | 0.647 | 0.934 |
|  | Kynurenine--oxoglutarate transaminase 3 | Weighted mode | 7 | 1.046(0.954-1.147） | 0.374 | 0.949 |
|  | Kynurenine--oxoglutarate transaminase 3 | Simple mode | 7 | 1.054(0.964-1.152） | 0.292 | 0.966 |
|  | Lactate | IVW | 7 | 0.998(0.991-1.005） | 0.59 | 0.983 |
|  | Lactate | MR Egger | 7 | 1.006(0.991-1.021） | 0.494 | 0.919 |
|  | Lactate | Weighted median | 7 | 0.999(0.991-1.007） | 0.773 | 0.934 |
|  | Lactate | Weighted mode | 7 | 0.999(0.989-1.01） | 0.93 | 0.986 |
|  | Lactate | Simple mode | 7 | 0.999(0.986-1.012） | 0.898 | 0.966 |
|  | Monounsaturated fatty acids | IVW | 7 | 1.003(0.995-1.011） | 0.484 | 0.983 |
|  | Monounsaturated fatty acids | MR Egger | 7 | 1.007(0.988-1.026） | 0.517 | 0.919 |
|  | Monounsaturated fatty acids | Weighted median | 7 | 1.001(0.993-1.009） | 0.797 | 0.934 |
|  | Monounsaturated fatty acids | Weighted mode | 7 | 1(0.989-1.011） | 0.948 | 0.986 |
|  | Monounsaturated fatty acids | Simple mode | 7 | 0.999(0.985-1.013） | 0.841 | 0.966 |
|  | Polyunsaturated fatty acids | IVW | 7 | 1.001(0.989-1.014） | 0.875 | 0.983 |
|  | Polyunsaturated fatty acids | MR Egger | 7 | 0.996(0.967-1.026） | 0.797 | 0.954 |
|  | Polyunsaturated fatty acids | Weighted median | 7 | 0.994(0.987-1.002） | 0.162 | 0.934 |
|  | Polyunsaturated fatty acids | Weighted mode | 7 | 0.993(0.984-1.003） | 0.205 | 0.949 |
|  | Polyunsaturated fatty acids | Simple mode | 7 | 0.995(0.984-1.005） | 0.371 | 0.966 |
|  | Retinol | IVW | 7 | 1.003(0.996-1.01） | 0.44 | 0.983 |
|  | Retinol | MR Egger | 7 | 1(0.984-1.016） | 0.979 | 0.979 |
|  | Retinol | Weighted median | 7 | 1.004(0.994-1.014） | 0.4 | 0.934 |
|  | Retinol | Weighted mode | 7 | 1.006(0.992-1.021） | 0.428 | 0.949 |
|  | Retinol | Simple mode | 7 | 1.006(0.992-1.02） | 0.412 | 0.966 |
|  | Total bilirubin | IVW | 7 | 0.996(0.981-1.012） | 0.647 | 0.983 |
|  | Total bilirubin | MR Egger | 7 | 1.004(0.969-1.04） | 0.822 | 0.954 |
|  | Total bilirubin | Weighted median | 7 | 0.991(0.972-1.01） | 0.336 | 0.934 |
|  | Total bilirubin | Weighted mode | 7 | 0.985(0.955-1.015） | 0.361 | 0.949 |
|  | Total bilirubin | Simple mode | 7 | 0.981(0.951-1.012） | 0.279 | 0.966 |
|  | Vitamin C | IVW | 7 | 1.001(0.993-1.008） | 0.879 | 0.983 |
|  | Vitamin C | MR Egger | 7 | 1.008(0.992-1.024） | 0.36 | 0.91 |
|  | Vitamin C | Weighted median | 7 | 1.003(0.994-1.011） | 0.561 | 0.934 |
|  | Vitamin C | Weighted mode | 7 | 1.003(0.992-1.014） | 0.646 | 0.986 |
|  | Vitamin C | Simple mode | 7 | 1.002(0.991-1.014） | 0.703 | 0.966 |
|  | Vitamin E | IVW | 7 | 0.998(0.99-1.005） | 0.518 | 0.983 |
|  | Vitamin E | MR Egger | 7 | 0.991(0.976-1.007） | 0.31 | 0.91 |
|  | Vitamin E | Weighted median | 7 | 0.994(0.985-1.004） | 0.238 | 0.934 |
|  | Vitamin E | Weighted mode | 7 | 0.996(0.983-1.008） | 0.524 | 0.949 |
|  | Vitamin E | Simple mode | 7 | 0.995(0.982-1.009） | 0.542 | 0.966 |
|  | Zinc | IVW | 5 | 1.001(0.953-1.05） | 0.983 | 0.983 |
|  | Zinc | MR Egger | 5 | 1.072(0.979-1.175） | 0.231 | 0.91 |
|  | Zinc | Weighted median | 5 | 1.027(0.978-1.08） | 0.287 | 0.934 |
|  | Zinc | Weighted mode | 5 | 1.03(0.964-1.1） | 0.433 | 0.949 |
|  | Zinc | Simple mode | 5 | 1.025(0.942-1.114） | 0.599 | 0.966 |

OSIB, oxidative stress injury biomarker; MR,mendelian randomization; SNP, single nucleotide polymorphism; IVW inverse-variance weighted; OR, odds ratio; CI, confidence interval.

| **Table S6. Heterogeneity and horizontal pleiotropy of lung cancer and 16 OSIBs.** | | | | | |
| --- | --- | --- | --- | --- | --- |
| Exposure | Outcome | Heterogeneity (MR Egger) | Heterogeneity (IVW) | Egger intercept | Pleiotropy |
|  |  | p_value | p_value |  | p_value |
| Squamous | Gamma-tocopherol | 0.244 | 0.138 | -0.016 | 0.397 |
|  | Hypoxanthine | 0.173 | 0.251 | 0.004 | 0.612 |
|  | Zinc | 0.904 | 0.347 | 0.042 | 0.385 |
|  | Kynurenine | 0.399 | 0.230 | -0.005 | 0.376 |
|  | Albumin | 0.371 | 0.440 | -0.001 | 0.740 |
|  | Lactate | 0.520 | 0.307 | 0.006 | 0.072 |
|  | Monounsaturated fatty acids | 0.244 | 0.213 | 0.004 | 0.257 |
|  | Polyunsaturated fatty acids | 0.845 | 0.838 | 0.003 | 0.361 |
|  | Glutathione peroxidase 7 | 0.437 | 0.511 | -0.006 | 0.759 |
|  | Glutathione S-transferase A1 | 0.849 | 0.889 | -0.006 | 0.735 |
|  | Catalase | 0.759 | 0.821 | 0.002 | 0.906 |
|  | Kynurenine--oxoglutarate transaminase 3 | 0.467 | 0.530 | 0.009 | 0.634 |
|  | Retinol | 0.599 | 0.648 | 0.002 | 0.556 |
|  | Vitamin C | 0.632 | 0.652 | -0.003 | 0.419 |
|  | Vitamin E | 0.647 | 0.473 | -0.007 | 0.108 |
|  | Total bilirubin | 0.248 | 0.288 | -0.005 | 0.564 |
| Adenocarcinoma | Gamma-tocopherol | 0.433 | 0.595 | 0.005 | 0.632 |
|  | Hypoxanthine | 0.057 | 0.078 | -0.005 | 0.636 |
|  | Zinc | 0.091 | 0.234 | 0.010 | 0.915 |
|  | Kynurenine | 0.685 | 0.238 | 0.005 | 0.348 |
|  | Albumin | 0.341 | 0.420 | 0.002 | 0.707 |
|  | Lactate | 0.617 | 0.711 | -0.001 | 0.895 |
|  | Monounsaturated fatty acids | 0.271 | 0.119 | 0.009 | 0.103 |
|  | Polyunsaturated fatty acids | 0.460 | 0.389 | 0.006 | 0.216 |
|  | Glutathione peroxidase 7 | 0.266 | 0.351 | 0.001 | 0.971 |
|  | Glutathione S-transferase A1 | 0.164 | 0.186 | -0.023 | 0.485 |
|  | Catalase | 0.915 | 0.948 | 0.007 | 0.787 |
|  | Kynurenine--oxoglutarate transaminase 3 | 0.453 | 0.533 | -0.012 | 0.658 |
|  | Retinol | 0.099 | 0.142 | -0.002 | 0.831 |
|  | Vitamin C | 0.199 | 0.023 | 0.017 | 0.041 |
|  | Vitamin E | 0.759 | 0.710 | 0.007 | 0.286 |
|  | Total bilirubin | 0.503 | 0.531 | -0.011 | 0.421 |
| Small cell lung cancer | Gamma-tocopherol | 0.739 | 0.855 | 0.004 | 0.720 |
|  | Hypoxanthine | 0.687 | 0.003 | -0.018 | 0.069 |
|  | Zinc | 0.339 | 0.168 | -0.080 | 0.196 |
|  | Kynurenine | 0.055 | 0.063 | -0.004 | 0.548 |
|  | Albumin | 0.297 | 0.413 | 0.000 | 0.990 |
|  | Lactate | 0.142 | 0.112 | -0.008 | 0.315 |
|  | Monounsaturated fatty acids | 0.021 | 0.032 | -0.004 | 0.673 |
|  | Polyunsaturated fatty acids | 0.000 | 0.000 | 0.005 | 0.719 |
|  | Glutathione peroxidase 7 | 0.670 | 0.690 | -0.029 | 0.439 |
|  | Glutathione S-transferase A1 | 0.249 | 0.343 | 0.013 | 0.765 |
|  | Catalase | 0.395 | 0.417 | -0.032 | 0.396 |
|  | Kynurenine--oxoglutarate transaminase 3 | 0.037 | 0.049 | 0.031 | 0.578 |
|  | Retinol | 0.667 | 0.758 | 0.003 | 0.685 |
|  | Vitamin C | 1.000 | 0.980 | -0.008 | 0.339 |
|  | Vitamin E | 0.484 | 0.501 | 0.007 | 0.392 |
|  | Total bilirubin | 0.209 | 0.277 | -0.008 | 0.645 |

OSIB, oxidative stress injury biomarker; MR,mendelian randomization; IVW, inverse-variance weighted.
