# Supplementary figures and images for: Deciphering bidirectional causal links between oxidative stress and lung cancer risk through Mendelian randomization
Source: Discov Oncol. 2025 Jul 28;16:1421. doi: 10.1007/s12672-025-03289-2 (PMC12304326; doi:10.1007/s12672-025-03289-2)

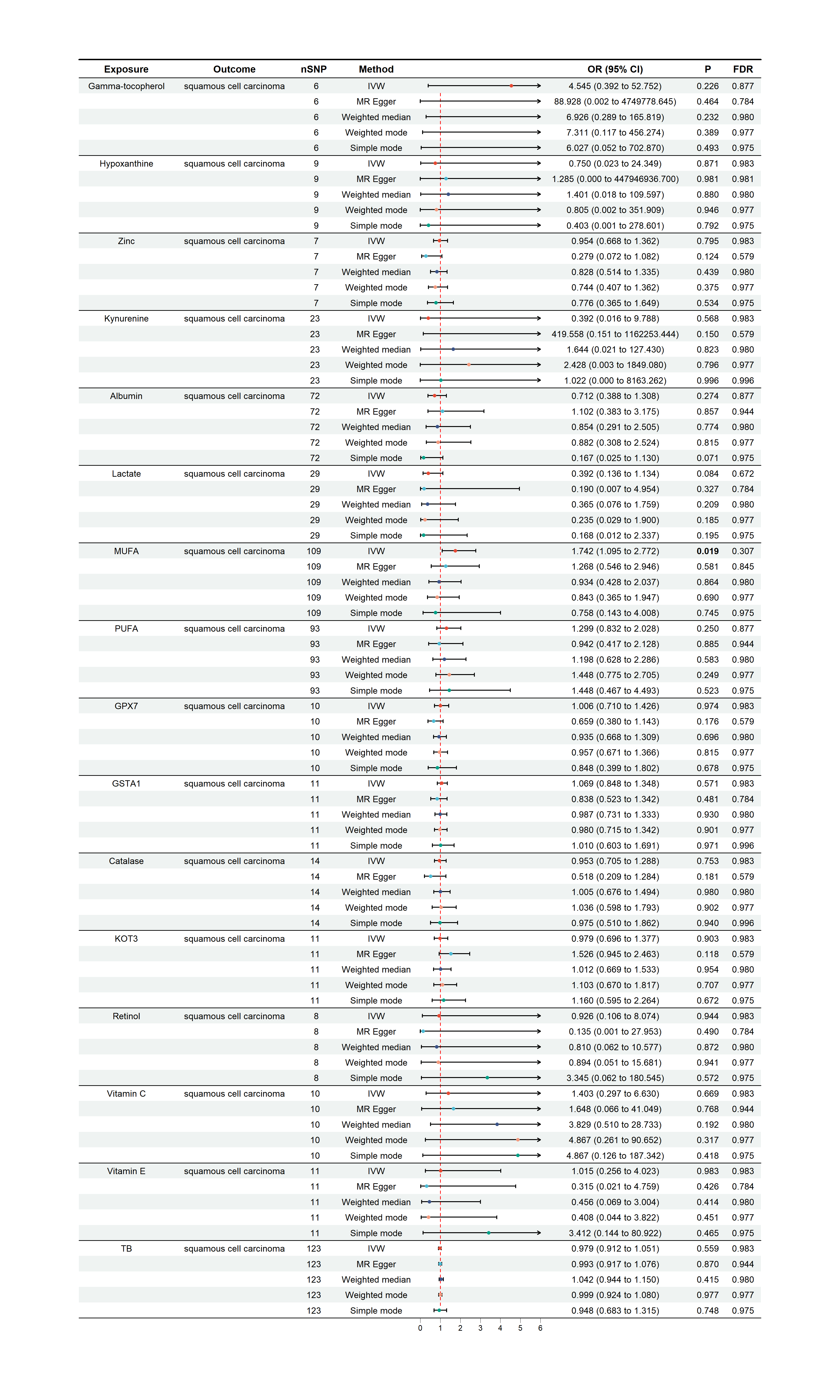

Supplement: Supplementary file 1 — Supplementary Material 1 [file 12672_2025_3289_MOESM1_ESM.zip › Supplementary materials/Figure S1.tiff]

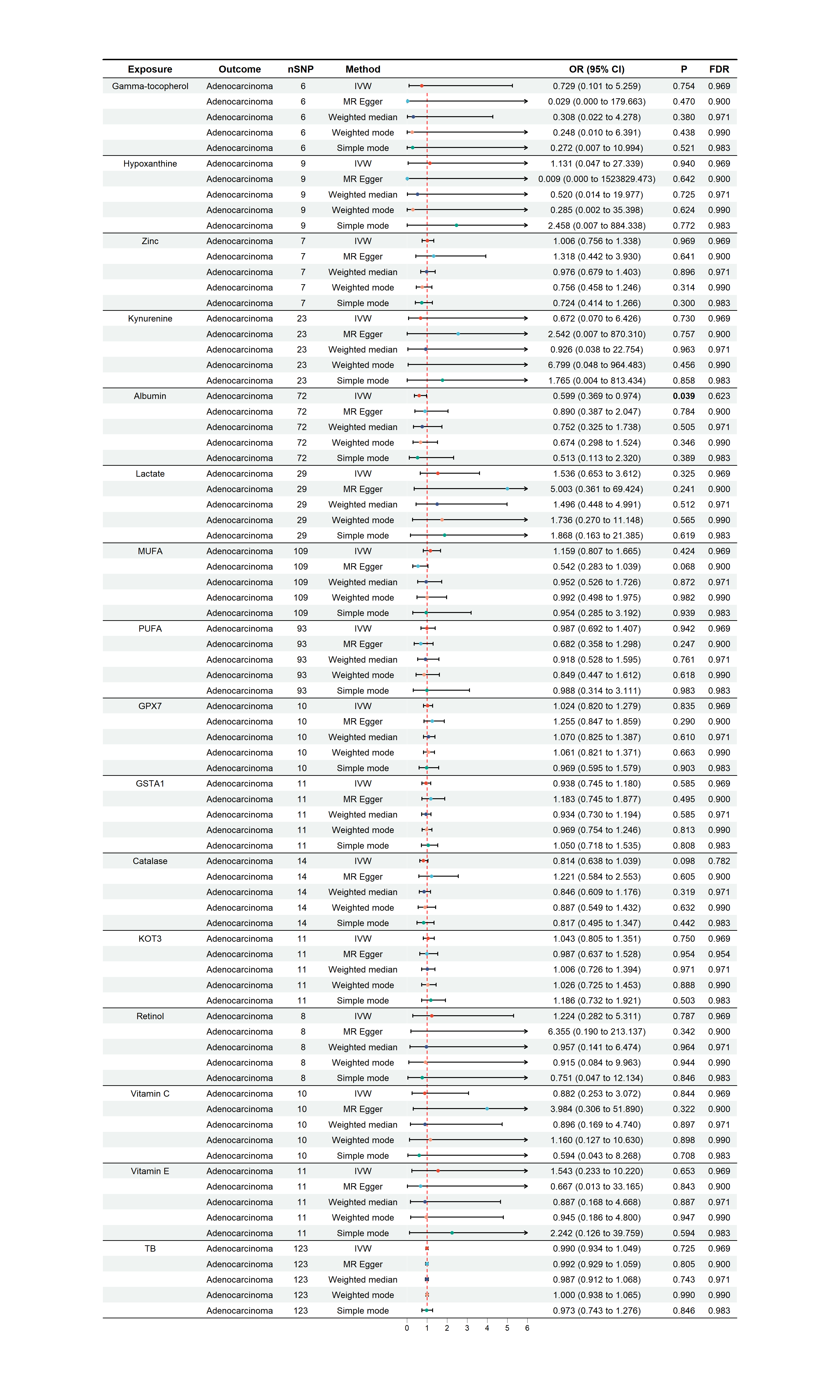

Supplement: Supplementary file 1 — Supplementary Material 1 [file 12672_2025_3289_MOESM1_ESM.zip › Supplementary materials/Figure S2.tiff]
